# Novel *Xanthomonas* Species From the Perennial Ryegrass Seed Microbiome – Assessing the Bioprotection Activity of Non-pathogenic Relatives of Pathogens

**DOI:** 10.3389/fmicb.2020.01991

**Published:** 2020-08-26

**Authors:** Tongda Li, Ross Mann, Timothy Sawbridge, Jatinder Kaur, Desmond Auer, German Spangenberg

**Affiliations:** ^1^Agriculture Victoria, AgriBio, Centre for AgriBioscience, Bundoora, VIC, Australia; ^2^DairyBio, Bundoora, VIC, Australia; ^3^School of Applied Systems Biology, La Trobe University, Bundoora, VIC, Australia

**Keywords:** bioprotection, *Xanthomonas*, perennial ryegrass, microbiome, non-pathogenic, secondary metabolite

## Abstract

The productivity of the Australian dairy industry is underpinned by pasture grasses, and importantly perennial ryegrass. The performance of these pasture grasses is supported by the fungal endophyte *Epichloë* spp. that has bioprotection activities, however, the broader microbiome is not well characterized. In this study, we characterized a novel bioprotectant *Xanthomonas* species isolated from perennial ryegrass (*Lolium perenne* L. cv. Alto). *In vitro* and *in planta* bioassays against key fungal pathogens of grasses (*Sclerotium rolfsii*, *Drechslera brizae* and *Microdochium nivale*) indicated strong bioprotection activities. A complete circular chromosome of ∼5.2 Mb was generated for three strains of the novel *Xanthomonas* sp. Based on the 16S ribosomal RNA gene, the strains were closely related to the plant pathogen *Xanthomonas translucens*, however, comparative genomics of 22 closely related xanthomonad strains indicated that these strains were a novel species. The comparative genomics analysis also identified two unique gene clusters associated with the production of bioprotectant secondary metabolites including one associated with a novel nonribosomal peptide synthetase and another with a siderophore. The analysis also identified genes associated with an endophytic lifestyle (e.g., Type VI secretion system), while no genes associated with pathogenicity were identified (e.g., Type III secretion system and effectors). Overall, these results indicate that these strains represent a novel, bioactive, non-pathogenic species of the genus *Xanthomonas*. Strain GW was the designated type strain of this novel *Xanthomonas* sp.

## Introduction

In Australia, the dairy industry has a farmgate value of $4.4 billion (2018 – 2019) and is ranked fourth for global market share (Dairy Australia, 2019). Despite its complexity in operation, the dairy industry can be summarized as the conversion of pastures grasses to milk and other dairy products. As such, the Australian dairy industry is underpinned by the performance of pasture grasses, and importantly perennial ryegrass.

The productivity of pasture grasses can be severely affected by plant pathogens. The major bacterial grass pathogen globally is *Xanthomonas translucens* pv. *graminis*, which causes bacterial wilt of pasture grasses (Egli and Schmidt, 1982), however, this pathovar is not present in Australia. According to the Victorian Plant Pathogen Herbarium (VPRI, Bundoora, Victoria, Australia), fungal grass pathogens are more common in Australia, including *Pyrenophora* spp., *Sclerotium* spp., *Phoma* spp., *Bipolaris* spp. and *Microdochium nivale*. The successful management of these pathogens is important for improving pasture productivity.

Biological controls (or biopesticides) are one management strategy that uses living organisms (e.g., microorganisms) to suppress deleterious or pathogenic organisms (Bulgarelli et al., 2013a). These bioprotection agents represent around 6.8% of the global pesticides market (2016) and are predicted to be worth $79.3 billion by 2022 (Chen, 2018). This growing area has seen more than 1320 bioprotection products registered in the US Environmental Protection Agency in 2014 (Mehrotra et al., 2017). For example, the fungal endophyte *Epichloë* spp. is a biological control that protects pasture grasses from herbivore via the production of bioactive compounds (Kauppinen et al., 2016). In addition, many bacteria have bioprotection activities, including *Bacillus* spp. and *Pseudomonas* spp. (Berg, 2009).

The plant microbiome provides an excellent reservoir where potential microbial bioprotection agents could be discovered. The diverse range of microorganisms associated with plant (plant microbiome) play a remarkable role in determining the health and productivity of the host (Berendsen et al., 2012). Therefore, substantial attention has been put on studying the bioprotection activities of these microorganisms (Bulgarelli et al., 2013b).

Next-generation sequencing technologies have led to fundamental changes to bacterial genomics by lowering cost and increasing throughput (Metzker, 2010). Recent advances in long read sequencing platforms like Oxford Nanopore Technologies (ONT) have made generating complete circular genomes for bacteria much easier (Koren et al., 2017). The availability of complete genome sequences underpins both the taxonomic identification and characterisation of novel microbial bioprotection agents, including the putative mode of action (i.e., identification of secondary metabolite gene clusters) and non-pathogenicity (i.e., absence of pathogenicity factors).

To gain insight into the broader microbiome of pasture grasses, we have profiled the microbiome of perennial ryegrass (*Lolium perenne* L. cv. Alto) and isolated bacterial strains (Tannenbaum et al., 2020), which were assessed for their beneficial activities (e.g., bioprotection). Three closely related strains (strain GW, seed-associated; strain SS and SI, mature-plant associated) exhibited excellent bioprotection activities against phytopathogens (*in vitro* and *in planta*). Complete genome assemblies were generated for these bacteria, and genome analysis showed that they represent a novel species of the genus *Xanthomonas*. Further bioinformatics analysis was conducted to determine the production of secondary metabolites that are putatively associated with bioprotection activities and to examine the presence/absence of pathogenicity related genes.

## Materials and Methods

### Bacterial Strain Isolation

Bacterial strains were isolated from perennial ryegrass (*Lolium perenne* L. cv. Alto, Barenbrug Agriseeds NZ). To isolate seed-associated bacteria, surface-sterilized seeds (3% NaOCl for 3 min, followed by 3 × sterile dH_2_O washes) were germinated under sterile conditions (on moistened sterile filter paper in sealed Petri dish). Germinated seedlings (5–7 days old) were harvested and sectioned into aerial and root tissue. Tissues were suspended in sterile Phosphate Buffered Saline (PBS), and ground using a Qiagen TissueLyser II (2 × 1 min at 30 Hz). Plant macerates were serial diluted (1:10, 100 μL in 900 μL), and plated onto Reasoners 2 Agar (R2A, Oxoid or Amyl Media, Australia) to isolate pure separated colonies. To isolate mature plant-associated bacteria, plants were grown in pots in a glasshouse for at least 60 days with standard potting mix and harvested for leaf and root tissues. Root tissues were washed in PBS to remove soil particles and then sonicated for 10 min to remove soil particulates and the rhizosphere. Tissue maceration, serial dilutions and bacterial isolations were prepared as above. All isolated bacterial strains were taxonomically classified using matrix assisted laser desorption ionization time-of-flight mass spectrometry (Bruker ultrafleXtreme MALDI-TOF/TOF MS and Biotyper System) (Tannenbaum et al., 2020), and stored in nutrient broth with 15% glycerol (v/v) at −80°C.

### Bioprotection Assay (*in vitro*)

An assay was designed to assess the *in vitro* bioprotection activity of bacterial strains against fungal phytopathogens of *Poaceae* species. The bacterial strains assessed included three xanthomonads (GW, SS, SI) and one *Paenibacillus* sp. (BU). Six fungal phytopathogens of *Poaceae* species (Supplementary Table S1) were obtained from the Victorian Plant Pathogen Herbarium (VPRI, Bundoora, VIC, Australia). Each bacterial strain was cultured in Nutrient Broth (BD Bioscience) overnight (OD = 1.0) and drop-inoculated (20 μL) onto four equidistant points on a Nutrient Agar (BD Bioscience) plate, which was then incubated overnight at 28°C. Then, a 6 × 6 mm plug of the phytopathogen (actively growing hyphae) was placed at the center of the plate and incubated at 28°C in dark. The incubation time varied to accommodate the differences in growth rate of the fungal pathogens (Table 1). The diameter of the fungal colony on the plate was measured twice. One reading was taken from the straight line that was defined by two inoculation points and the center of the plate, and the other reading was taken after rotating the plate for 45 degrees. The average of the two readings was used for statistical analysis. For each treatment, three plates were prepared as biological replicates. For the blank control, sterile Nutrient Broth was used to replace the bacteria. Statistical analysis (One-way ANOVA and Tukey Test) was conducted using OriginPro 2018 (Version b9.5.1.195) for any significant difference (*P* < 0.05) between treatments.

**TABLE 1:** The average colony diameter (±standard error) of fungal pathogens when exposed to the three xanthomonads in a bioprotection assay (*in vitro*).

**Pathogen ID**	**T_*i*__*ncubation*_/day**	**GW/cm**	**BU/cm**	**Blank/cm**	**SS/cm**	**SI/cm**	**Blank/cm**
*P. sorghina*	9	2.83 ± 0.12^a^	3.90 ± 0.06^b^	4.43 ± 0.07^b^	N/A	N/A	N/A
*D. brizae*	8	3.13 ± 0.07^a^	3.67 ± 0.03^b^	3.90 ± 0.06^b^	2.63 ± 0.30^a^	2.33 ± 0.42^a^	4.50 ± 0.21^b^
*S. rolfsii*	5	2.13 ± 0.14^a^	6.10 ± 0.10^b^	8.47 ± 0.03^c^	2.13 ± 0.27^a^	1.87 ± 0.14^a^	8.46 ± 0.03^b^
*B. gossypina*	7	2.27 ± 0.24^a^	3.07 ± 0.07^a^	5.00 ± 0.12^b^	6.08 ± 0.22^a^	5.95 ± 0.05^a^	7.05 ± 0.41^a^
*F. verticillioides*	10	4.67 ± 0.07^a^	6.47 ± 0.09^b^	6.90 ± 0.25^b^	5.03 ± 1.09^a^	6.43 ± 0.72^a^	7.97 ± 0.03^a^
*M. nivale*	6	2.37 ± 0.18^a^	6.70 ± 0.12^b^	7.37 ± 0.07^b^	7.83 ± 0.12^a^	6.90 ± 1.05^a^	7.97 ± 0.03^a^

*^*a,b,c*^: Different letters are statistically significantly (*P* < 0.05) different. Strain GW/SS/SI: *Xanthomonas* sp. Strain BU: *Paenibacillus* sp.*

### Bioprotection Assay (*in planta*)

An assay was designed to assess the *in planta* bioprotection activity of the bacterial strains against the fungal phytopathogen *Bipolaris sorokiniana* (VPRI 42684). The xanthomonad strain GW was used in this assay. Wheat seeds were surface-sterilized as per section 2.1. The bacterial strain was cultured in Nutrient Broth (BD Bioscience) for 6 h (OD = 0.5). Sterile seeds were imbibed in the bacterial culture for 18 h, removed from the culture, dried under sterile conditions and then germinated in dark at room temperature (23°C) for 4 days for root and shoot development. Germinated seedlings were transferred into pots with standard potting mix (4 seeds per pot, 4 pots per treatment) in a glasshouse (Supplementary Table S2) for 39 days. A 7 cm segment of the lowest leaf that was green and fully extended from each plant was excised and placed on 0.5% water agar. A sterile sharp needle was used to create a wound at the center of each leaf, to which 1 μL of *B. sorokiniana* spore suspension (8.5 × 10^3^ spores/mL) was added. Plates were then sealed and left at room temperature (23°C) for 3 days. To assess the bioprotection activity, the size (measured in mm^2^) of the lesion, chlorotic zones and fungal hyphal growth was recorded. For the blank control, sterile Nutrient Broth was used. Statistical analysis (One-way ANOVA and Tukey Test) was conducted using OriginPro 2018 (Version b9.5.1.195) for any significant difference (*P* < 0.05) between treatments.

### Genome Sequencing

DNA was extracted from bacterial pellets of GW, SS and SI (overnight cultures) using a Wizard^®^ Genomic DNA Purification Kit (A1120, Promega, Madison, WI, United States), and assessed for quality (average molecular weight ≥ 30 Kb) on an Agilent 2200 TapeStation (Agilent Technologies, Santa Clara, CA, United States).

Genomic sequencing libraries (short reads) were prepared from the DNA using the Illumina Nextera XT DNA library preparation kit (Cat# FC-131-1096) and sequenced on an Illumina HiSeq 3000 platform. Genomic sequence data (raw reads) were assessed for quality and filtered to remove any adapter and index sequence, and low-quality bases using fastp (Chen et al., 2018) with the following parameters: *-w 8 -3 -5*.

Genomic sequencing libraries (long reads) were prepared from the DNA using the Oxford Nanopore Technologies (ONT) transposases-based library preparation kit with minor modifications (SQK-RAD004, ONT, Oxford, United Kingdom) and sequenced on a MinION Mk1B platform (MIN-101B) with R9.4 flow cells (FLO-MIN106). Genomic sequence data (raw read signals) were basecalled using ONT’s Albacore software (Version 2.3.4), and assessed for quality using NanoPlot (De Coster et al., 2018). Basecalled data was filtered to remove adapter sequences using Porechop (Version 0.2.3^1^), while reads shorter than 300 bp and the worst 5% of reads (based on quality) were discarded using Filtlong (Version 0.2.0^2^).

### Genome Assembly, Classification and Alignment

The whole genome of GW, SS, and SI were assembled with filtered long and short reads using Unicycler (Wick et al., 2017). Long reads were used for primary assembly and to resolve repeat regions in the genome, whereas short reads were used to correct small base-level errors. Assembly graphs were visualized using Bandage (Wick et al., 2015). Assembled genomes were taxonomically classified by Kraken2 (Wood and Salzberg, 2014) using a custom database containing all completed bacterial reference genomes in NCBI (20/03/2020). Genomes of GW, SS, and SI were aligned using LASTZ (Version 1.04.00^3^), and visualized using AliTV (Ankenbrand et al., 2017).

### Genome Annotation and Characterisation

The assembled genome of GW, SS and SI were annotated using Prokka (Seemann, 2014) with a custom *Xanthomonas* protein database (based on Kraken2 classification) to predict genes and corresponding functions. A further functional characterisation of annotated genomes was conducted using KEGG BlastKOALA (Kanehisa et al., 2016). Identification of secondary metabolite gene clusters from annotated genomes was conducted using antiSMASH (Weber et al., 2015) with the following options: *–clusterblast –asf –knownclusterblast –subclusterblast –smcogs –full-hmmer*. An evaluation of the presence of pathogenicity-related genes from the annotated genomes of all three strains (GW, SS, and SI) was conducted using BLAST (Camacho et al., 2009) (blastp and tblastn, *e*-value > 1e^–10^). Initially, pathogenicity-related genes previously reported in *Xanthomonas* spp. were targeted (133 genes), including secretion systems (Type I/II/III/VI), pili (Type IV), flagella, pathogenicity regulatory factors, xanthan biosynthesis and lipopolysaccharide biosynthesis. A further comparison of 36 genes involved in Type III secretion systems (T3SS) from six pathogenic strains, including *X. translucens* pv. *translucens* DSM18974, *X. translucens* pv. *undulosa* Xtu4699, *X. translucens* pv. *cerealis* CFBP2541, *X. translucens* DAR61454, *X. translucens* pv. *graminis* Xtg29 and *X. translucens* pv. *graminis* ICMP6431, and the three strains was conducted, including structural and regulatory genes, as well as conserved and variable Type III effectors (T3Es). Transcription activator-like effectors (TALEs) were predicted from the three strains (GW, SS and SI) and three pathogenic strains using annoTALE (Grau et al., 2016). Since TALE genes usually have multiple near-perfect repeats in the sequence and multiple copies of sequences in the genome (White et al., 2009), short reads often struggle to properly assemble the TALEs regions (Peng et al., 2016). Therefore, only pathogenic strains whose genome was completely assembled, i.e., *X. translucens* pv. *translucens* DSM18974, *X. translucens* pv. *undulosa* Xtu4699 and *X. translucens* pv. *cerealis* CFBP2541, were used in the prediction of TALE genes. The genome of strain GW and *X. translucens* pv. *undulosa* Xtu4699 were aligned using BLAST (Camacho et al., 2009). The alignment as well as the T3SS, T3Es and TALE genes that were detected on the genome of *X. translucens* pv. *undulosa* Xtu4699 were visualized using BRIG (Alikhan et al., 2011).

### Phylogeny and Comparative Genomics

Eighteen Group 1 *Xanthomonas* spp. genomes and one *X. campestris* genome (Group 2 *Xanthomonas*) that were publicly available on NCBI (Supplementary Table S3) were downloaded and used for phylogenetic analysis (Young et al., 2008). These genomes were annotated *de novo* using the method above. Genes that were shared by all strains were identified using Roary and aligned (codon aware) using PRANK (Löytynoja, 2014). A maximum-likelihood phylogenetic tree was inferred using FastTree (Price et al., 2010) with Jukes-Cantor Joins distances, the Generalized Time-Reversible substitution model and the CAT approximation model. Local branch support values were calculated using 1000 resamples with the Shimodaira–Hasegawa test.

## Results

### Bioprotection Assay (*in vitro*)

*Xanthomonas* sp. strain GW significantly (*P* < 0.05) reduced the average colony diameter of all six fungal pathogens compared to the blank control, and four pathogens compared to *Paenibacillus* sp. strain BU (Table 1). Strain GW reduced the growth of *S. rolfsii, M. nivae, D. brizae, P. sorghina, F. verticillioides* and *B. gossypina* by 74.9, 67.8, 54.6, 36.1, 32.3, and 19.7%, respectively, compared to the blank control. Strain SI reduced the growth of *S. rolfsii* and *D. brizae* by 77.9 and 48.2%, respectively, and strain SS reduced the growth of *S. rolfsii* and *D. brizae* by 74.8 and 41.6%, respectively, when compared to the blank control. When comparing across the three xanthomonads, only strain GW significantly inhibited the growth of all pathogens, indicating its broad-spectrum bioprotection activity (Supplementary Figures S1, S2).

### Bioprotection Assay (*in planta*)

**FIGURE 1:**
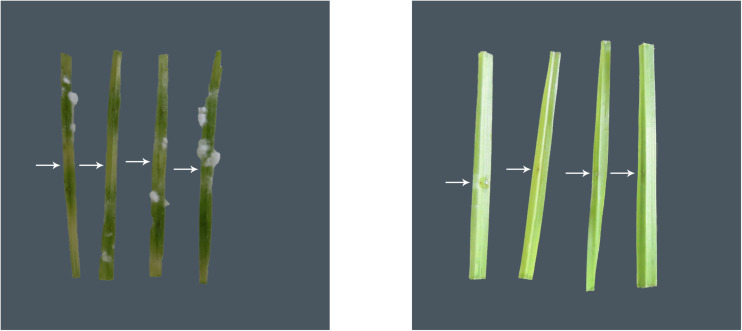
Representative images of the *in planta* bioprotection assay for the blank control group (left) and the treatment group (inoculated with strain GW, right), with white arrows representing the point of inoculation of the pathogen *B. sorokiniana* (VPRI 42684) in wheat. Extensive leaf discoloration and white fungal hyphal growth are seen away from the point of inoculation in the blank control leaves, but not in the GW inoculated leaves.

*Xanthomonas* sp. strain GW significantly (*P* < 0.05) reduced the average size of lesion and fungal hyphal growth compared to the blank control (Figure 1 and Table 2). The lesion size was reduced by 96.7%, and the area of fungal hyphal growth was reduced by 94.7%.

**TABLE 2:** The average size of area showing disease symptoms (±standard error) of *B. sorokiniana* when exposed to strain GW in a bioprotection assay (*in planta* in wheat).

**Strain ID**	**Lesion/mm^2^**	**Chlorosis/mm^2^**	**Fungal hyphal growth/mm^2^**
GW	1.33 ± 0.25^*a*^	34.44 ± 10.72^*a*^	2.00 ± 1.37^*a*^
Blank	42.75 ± 10.26^*b*^	68.88 ± 22.50^*a*^	37.63 ± 20.45^*b*^

*^*a, b*^: Different letters are statistically significantly (*P* < 0.05) different.*

### Genome Sequencing, Assembly and Annotation

A total of 9,674,929,775 bp short reads and 761,078,031 bp long reads were generated (Supplementary Table S4). Complete circular genome sequences were produced for all three strains. The genome size for strain GW, SS and SI were 5,233,349 bp (4358 CDSs), 5,185,085 bp (4227 CDSs) and 5,246,417 bp (4290 CDSs), respectively, (Table 3). The percent GC content ranged from 68.37% to 68.55%. There were no plasmids present in any strain.

**TABLE 3:** General genomic characteristics of the three *Xanthomonas* strains.

**Strain ID**	**Genome size (bp)**	**GC content (%)**	**No. of tRNA**	**No. of tmRNA**	**No. of rRNA**	**No. of gene**	**No. of CDS**
GW	5,233,349	68.37	60	1	6	4425	4358
SS	5,185,085	68.55	57	1	6	4291	4227
SI	5,246,417	68.44	63	1	6	4360	4290

### Phylogeny and Comparative Genomics

The three *Xanthomonas* strains (GW, SS, and SI) were phylogenetically related to *Xanthomonas translucens* (strain XT2, Genbank Accession: NR_036968.1) with a sequence coverage of 100% and homology of 99.53 – 99.73% based on the 16S ribosomal RNA gene. The genomes of the three xanthomonads were also classified as *X. translucens* pv. *cerealis* (NCBI:txid 152263) by Kraken2, suggesting their close relationship with *X. translucens*.

**FIGURE 2:**
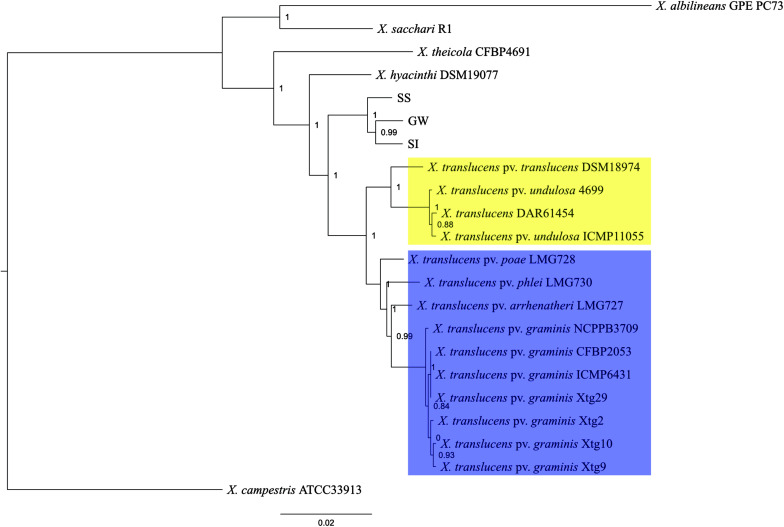
Phylogeny of Group 1 *Xanthomonas* species and strain GW, SS and SI. This maximum-likelihood tree was inferred based on 68 genes conserved among 22 genomes. Values shown next to branches were the local support values calculated using 1000 resamples with the Shimodaira–Hasegawa test. Strain GW, SS and SI formed a clade that was well separated from *X. translucens* pathovars that are pathogenic on crop species (yellow) and grass species (blue).

A comparative genomics analysis indicated that the three *Xanthomonas* strains (GW, SS, SI) belonged to the Group 1 *Xanthomonas* based on a sequence homology comparison of 68 genes shared by all 22 strains (Figure 2). The topology of the tree was consistent with Young et al. (2008), with unique clades/branches apparent for *X. albilineans*, *X. sacchari*, *X. theicola*, *X. hyacinthi* and *X. translucens*, with the three *Xanthomonas* strains (GW, SS, SI) between *X. hyacinthi* and *X. translucens*. The tree showed the three *Xanthomonas* strains (GW, SS, SI) formed a unique clade adjacent to *X. translucens* pathovars and were separated with a strong local support value (100%). The *X. translucens* clade were divided into a subclade consisting of *X. translucens* pv. *translucens* DSM18974, *X. translucens* pv. *undulosa* Xtu4699, *X. translucens* pv. *undulosa* ICMP11055 and *X. translucens* DAR61454 (Figure 2, yellow, barley and wheat pathogens) and a subclade consisting of *X. translucens* pv. *arrhenatheri* LMG727, *X. translucens* pv. *poae* LMG728, *X. translucens* pv. *phlei* LMG730 and all *X. translucens* pv. *graminis* strains (Figure 2, blue, pasture grass pathogens).

Average nucleotide identity (ANI) was calculated to further elucidate the relationship between the three *Xanthomonas* strains (GW, SS and SI) and *X. translucens* pathovars (Supplementary Table S5). The results showed 97.20 – 97.39% similarities between the three xanthomonads, and 92.97 – 94.07% similarities between the three xanthomonads and *X. translucens* pathovars.

### Pathogenicity-Related Gene Analysis

**FIGURE 3:**
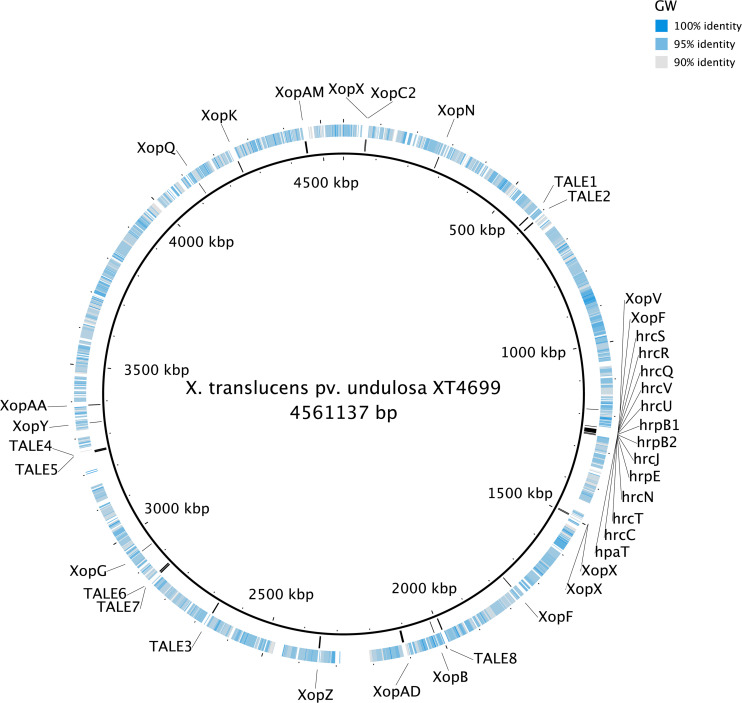
The genome alignment of the strain GW (the outer circle) and *X. translucens* pv. *undulosa* Xtu4699 (the inner circle, black). The color of the outer circle represented the sequence identity (gray to blue: 90–100%; white blocks: <90%) The locations of T3SS, T3Es and TALEs genes detected in the genome of *X. translucens* pv. *undulosa* Xtu4699 are also displayed.

The genomes of the three *Xanthomonas* strains (GW, SS and SI) were found to have a reduced complement of pathogenicity-related genes. The assessment of 133 pathogenicity-related genes identified that the three *Xanthomonas* strains (GW, SS and SI) was devoid of the T3SS that is critical for pathogenicity of most *Xanthomonas* species (White et al., 2009; Wichmann et al., 2013) (Supplementary Table S6). A comprehensive assessment of the T3SS structural and regulatory genes and T3Es across the three *Xanthomonas* strains (GW, SS and SI) and six pathogenic *X. translucens* strains identified that the three strains had 0 of 37 T3SS genes and T3Es (Table 4; Figure 3). This included an absence of the *hrc* genes, which encode the injectisome (Wagner et al., 2018), and the *hrp* genes, which are essential to suppress host plant defense responses for *Xanthomonas* species (Kay and Bonas, 2009). The *hrpF* gene, which encodes a translocon protein complex that is required to deliver T3Es (Chatterjee et al., 2013), was missing in all nine strains, which was supported by previous research (Pesce et al., 2017). However, the *hpaT* gene, which was described to encode an undescribed translocon protein complex of *X. translucens* strains (Pesce et al., 2017), was detected in all pathogenic strains but not in the three *Xanthomonas* strains (GW, SS and SI).

**TABLE 4:** T3SS and T3Es genes in the genome of the three *Xanthomonas* strains (GW, SS and SI) and other *X. translucens* strains.

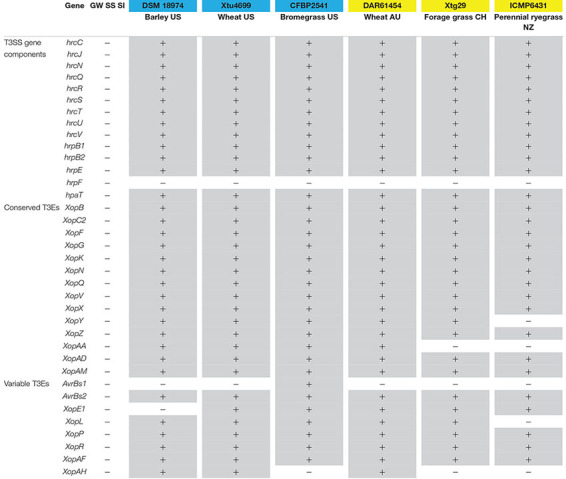

*Gray (+)/white (−): presence/absence of gene in genome. Blue: strains that have a complete genome sequence available, Yellow: strains that have no complete genome sequence available.*

Similar to the T3SS and T3Es genes, no TALE genes could be identified in the genome of the three *Xanthomonas* strains (GW, SS and SI). Eight TALE genes were predicted for strain *X. translucens* pv. *undulosa* Xtu4699 (Figure 3) and *X. translucens* pv. *translucens* DSM18974, and two TALE genes were predicted for strain *X. translucens* pv. *cerealis* CFBP2541.

### Secondary Metabolite

**FIGURE 4:**
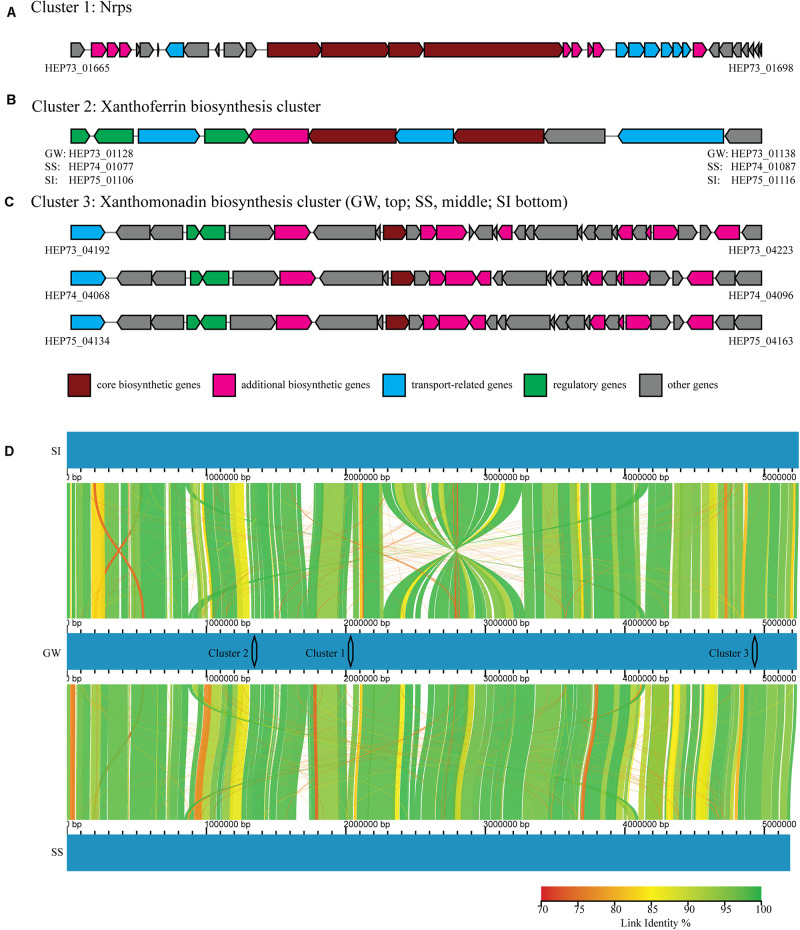
Three secondary metabolite gene clusters **(A–C)** identified by antiSMASH, including core biosynthetic genes (maroon), additional biosynthetic genes (pink), regulatory genes (green), transport-related genes (blue) and other genes (gray). The NCBI identifiers were shown for genes located at both ends of each cluster. **(D)** Whole genome comparison of strain GW (middle), SI (top) and SS (bottom), with color graduation representing nucleotide percentage similarity between genomes (from 70 to 100%, red to green). The locations of the three clusters were also represented.

The *in vitro* and *in planta* bioprotection activity of the three *Xanthomonas* strains (GW, SS and SI) indicated that they could produce biocidal secondary metabolites. Furthermore, it has been demonstrated that both live culture and cell-free extracts of strain GW have biocidal activity against fungal phytopathogens (unpublished data). Secondary metabolite gene analysis identified three clusters (Clusters 1 – 3), with strain GW having all three clusters, and strain SI and SS having two of the three clusters. These clusters contain all the genes (core/additional biosynthetic genes, regulatory genes, transport-related genes and other genes) required for complete function (Figures 4A–C).

Cluster 1 contained a nonribosomal peptide synthetase (Nrps), and the entire cluster was unique to strain GW. Cluster 1 was located between bases 1,997,794 and 2,067,075 in the genome of strain GW, while this region was absent from strain SS and SI (Figure 4D). Cluster 1 appears novel based on sequence homology searching against the antiSMASH gene clusters database. Cluster 2 contained a siderophore synthetase and the entire cluster was present in all three strains. Cluster 2 was located between bases 1,300,000 and 1,380,000 in the genomes of strain GW, SS and SI (Figure 4D). Cluster 2 has sequence homology to the xanthoferrin biosynthesis gene cluster. Cluster 3 contained an aryl polyene synthase and the entire cluster was present in all three strains, however, slight variations in the cluster structure were observed (Figure 4C). Cluster 3 was located between bases 4,860,000 and 4,980,000 in the genomes of strain GW, SS and SI (Figure 4D). Cluster 3 has sequence homology to the xanthomonadin biosynthesis gene cluster.

## Discussion

Plant microbiomes are a repository from which plant beneficial bacteria can be isolated and identified. In this study, we compared three related *Xanthomonas* strains from the *L. perenne* microbiome. These had differing *in vitro* bioprotection activities, and the strain with the strongest activities against a wide range of phytopathogens (GW) became the focus of this study. Based on the complete genome assembly, strain GW possesses a novel Nrps cluster compared to the other two strains. All three strains lack many of the genes that are essential for pathogenicity in pathogenic *Xanthomonas* strains. Such characteristics made strain GW a promising candidate to be developed as a bioprotection agent for crops and grasses.

### Identification of a Novel Xanthomonas Species

Taxonomic identification of bacterial species often uses 16S ribosomal RNA, whole genome sequence homology and ANI, with each technique providing varying degrees of taxonomic resolution. Taxonomic assignment based on the 16S ribosomal RNA gene provides genus level resolution, whereas whole genome techniques provide species or sub-species resolution. In this study, the initial classification based on 16S ribosomal RNA and whole genome sequence against the NCBI RefSeq database suggested that strain GW, SS and SI were most likely representatives of the plant pathogenic *X. translucens*. However, comparative genome analysis demonstrated the three strains formed a cluster that was separated from other *X. translucens* pathovars. Most importantly, the ANI between these three strains and *X. translucens* pathovars was lower than the species boundary, which is 95 – 96% ANI (Richter and Rosselló-Móra, 2009; Chun et al., 2018). Therefore, the three xanthomonads used in this study represent a novel species of the genus *Xanthomonas*. This clearly demonstrated the limitations of 16S ribosomal RNA-based classification (Klenk and Goker, 2010). Due to the technical limitations of the short-read sequencing platforms, most microbiome studies only used a variable region of the 16S ribosomal RNA (Pollock et al., 2018). It was likely that such novel, bioactive *Xanthomonas* species were present in the samples but were overlooked since they were classified as the known pathogenic *Xanthomonas* species. Moreover, this study also emphasized the importance of available whole genome sequences. The hybrid assembly approach used here combined the advantages of both short reads and long reads to produce high quality genome sequences for all three strains, which underpinned the downstream analysis including taxonomic identification and functional characterisation of the genomic resources.

### Absence of Pathogenicity-Related Genes in the Three Xanthomonads

An analysis of pathogenicity-related genes clarified that the three xanthomonads were highly likely non-pathogenic. The T3SS and T3SS-related effector proteins (T3Es and TALEs) were completely absent from the genome of the three xanthomonads. The T3SS is a needle and syringe-like system that delivers (i) T3Es that suppress plant innate immunity and modulate plant cellular pathways to enhance bacterial infection (Büttner, 2016), and (ii) TALEs that induce host susceptibility genes to enhance virulence (Cernadas et al., 2014; Hu et al., 2014), both of which are important for pathogenicity in *Xanthomonas* species (Green and Mecsas, 2016). For instance, deletion mutations of the T3SS structural genes *hrpE* or *hrcR* showed significantly reduced symptoms of *X. translucens* pv. *graminis* Xtg29 when compared with the wildtype strain (Wichmann et al., 2013). Furthermore, complete loss of symptoms was observed for a *X. translucens* pv. *undulosa* Xtu4699 strain with an insertion mutation in the T3SS structure gene *hrcC* (Peng et al., 2016). Complete absence of the T3SS and T3Es has been reported in other *Xanthomonas* species, such as *X. arboricola* strains (Group 2 *Xanthomonas*) which were referred to as non-pathogenic (Garita-Cambronero et al., 2017).

It must be stated that some xanthomonads that lacked the Hrp T3SS were found to be associated with diseased plants including *X. cannabis* NCPPB3735 and *X. cannabis* NCPPB2877 strains (Group 2 *Xanthomonas*) that could cause symptoms on hemp, barley and tobacco (Jacobs et al., 2015). While they lacked the Hrp T3SS, they had *HrpG* and *HrpX*, which are two key Hrp pathogenicity regulator genes (Büttner and Bonas, 2010), that were absent from the genome of the three xanthomonads. Moreover, there is another pathogenic strain of the same species, *X. cannabis* pv. *phaseoli* (Nyagatare strain), that has been reported to have both regulator genes, the full Hrp T3SS and T3Es (Aritua et al., 2015). In Group 1 *Xanthomonas*, *X. albilineans* GPE PC73, which is a xylem-limited pathogen, also lacked the Hrp T3SS (Pieretti et al., 2009). However, this strain had a *Salmonella* pathogenicity island-1 (SPI-1) containing an alternate T3SS and a gene cluster that encodes the phytotoxic albicidin, neither of which was detected in the three xanthomonads used in this study. *X. sacchari*, which is also a Group 1 *Xanthomonas*, lacked the Hrp T3SS and the SPI-1 T3SS (Studholme et al., 2011). However, the strain was isolated from an insect from a diseased banana plant and there was no evidence of plant pathogenicity, which could be explained by the missing T3SS. T3SS has been proven to be essential for pathogenicity for *X. translucens* (Wichmann et al., 2013), which has the closest phylogenetic relationship amoug all known *Xanthonomas* species to the three xanthomonads in this study. Therefore, without any known type of T3SS, T3Es and TALEs the three xanthomonads are highly likely non-pathogenic, and no symptoms have been seen in inoculated wheat, barley and ryegrass plants. Furthermore, given the fact that these genes are widely distributed across the whole chromosome (Figure 3), they are highly unlikely to acquire all the genes necessary to become pathogenic through horizontal gene transfer.

The three xanthomonads contained gene clusters (T1SS, T2SS, T6SS, type 4 pilus, flagella) linked to pathogenicity of *X. translucens* pathovars (Supplementary Table S6), however, these clusters have also been reported to possess functions associated with an endophytic lifestyle. For example, the T1SS was associated with biofilm formation (Tseng et al., 2009), the T2SS was used to secrete enzymes that facilitate environmental adaptation (Green and Mecsas, 2016), and the T6SS was involved in communication between bacteria or bacteria and the symbiotic host plant (Boyer et al., 2009). The three xanthomonads also had a type IV pilus cluster and a flagellar gene cluster that are associated with adherence and motility (Dunger et al., 2016; Hersemann et al., 2017). The presence of these gene clusters is supportive of the endophytic lifestyle proposed for the three xanthomonads.

### Bioprotection Activity and Putative Mode of Action

Biological controls agents (e.g., bioprotectant bacteria) have been widely adopted globally for managing plant diseases as they are an effective and environmentally sustainable alternative to agrochemicals (Glare et al., 2012). For instance, biological control agents offer unique, complex modes of action, whereas agrichemicals have specific mode of action that can more easily lead to the development of resistance (Grimmer et al., 2015). Furthermore, there is less regulatory burden associated with biological control agents, in contrast to some agrichemicals that are under increased regulatory scrutiny as they have increasing environmental and public health concerns (Bach et al., 2016; Droby et al., 2016). Many *Bacillus-* and *Pseudomonas*- based biological control products have been commercialized globally for controlling bacterial and fungal phytopathogens (e.g., *Bacillus subtilis* for controlling *Fusarium* spp., and *Pseudomonas fluorescens* for controlling *Erwinia amylovora*) (Berg, 2009). These types of bacteria protect plants from phytopathogens directly via microbial antagonism, either endophytically (within the plant) or on the rhizosphere and phyllosphere (on the plant surface) (Eljounaidi et al., 2016; O’brien, 2017). Such antagonism can be carried out by competing for nutrients and spaces for colonization on the plant surface (Kamilova et al., 2005), and/or synthesizing allelochemicals such as antibiotics and siderophores to suppress pathogens (Beneduzi et al., 2012).

Bioprotection activity against fungi has not been reported to be associated with xanthomonads. *Xanthomonas* spp. are commonly associated with plants as either endophytes (Bouffaud et al., 2014; Bulgarelli et al., 2015; Zarraonaindia et al., 2015; Mitter et al., 2017) or as phytopathogens (An et al., 2019). In this study, we isolated three xanthomonads that had bioprotection activity, providing inhibitory activity against fungal pathogens from a broad taxonomic range (2 Phyla, 5 Families) in *in vitro* and *in planta* assays. The activity observed in the assays was a reduction in growth of the pathogen, and while no complete control was observed the xanthomonad strains restricted growth up to 77.9% of some pathogens. Strain GW also provided prolonged protection (up to 39 days) against the pathogen in the *in planta* assay. This suggests two methods of plant protection including (1) localized microbial colonization of a plant tissue from which antibiotic compounds are produced that are translocated systemically throughout the plant, or (2) systemic microbial colonization of the plant from which the bacteria either competes for nutrients or produces antibiotic compounds. Method 1 is utilized by *Epichloë* spp. endophytes to protect *Poaceae* species against pests and pathogens (Johnson et al., 2013), whereas method 2 is utilized by *Erwinia* and *Pantoea* species (Born et al., 2016; Smits et al., 2019). Given the *in vitro* bioprotection assay indicated production of a bioactive suppressant, we propose that the *in planta* activity is analogous. Further experiments including *in planta* assays and glasshouse and field assays have been planned to explore the potential bioactivity of strain GW.

*Xanthomonas* spp. have been shown to produce an array of bioactive secondary metabolites including the siderophore xanthoferrin, the pigment xanthomonadin, and the polysaccharide xanthan gum (Poplawsky et al., 2000; He et al., 2011; Palaniraj and Jayaraman, 2011; Huang et al., 2015; Pandey et al., 2017; Madden et al., 2019). A genomics-based assessment identified two secondary metabolite gene clusters that could be linked to the bioactivity of strain GW, SS and SI. A xanthoferrin siderophore synthesis cluster was detected in all three strains. First described in *X. campestris* pv. *campestris*, xanthoferrin is a vibrioferrin-type siderophore which facilitate iron uptake of bacteria by binding ferric iron from the environment (Andrews et al., 2003). Bacterial siderophores have higher affinity to iron compared to fungal siderophores (Compant et al., 2005), and therefore they can act as bioprotection agents under iron-limiting environments by depriving fungi of this essential element. This has been observed in fluorescent pseudomonads against the fungal pathogen *Fusarium oxysporum* (Kloepper et al., 1980; Dwivedi and Johri, 2003). Therefore, xanthoferrin could be responsible for the *in vitro* bioprotection activity that was observed from the three xanthomonads. Given that siderophores are predominantly produced locally (Saha et al., 2016), the mode of action of such bioprotection activity could be explained by method 2 described above. Moreover, strain GW showed a stronger and broader spectrum bioprotection activity against phytopathogens compared to strain SS and SI. Given that a novel Nrps cluster that was unique to strain GW but was missing from strain SS and SI, we proposed a hypothesis that the product of this novel Nrps cluster was responsible for the broad-spectrum of bioprotection activity of strain GW. Further research is needed to prove this hypothesis, including creating mutants of the Nrps cluster and evaluate the bioprotection activity (*in vitro*), along with identifying, purifying and characterizing the active compound(s). The mode of action of the bioprotection activity that provide by this Nrps could be either method described above.

## Data Availability Statement

Annotated genome sequences of all strains were deposited in the NCBI GenBank with the accession numbers: CP051189 for GW, CP051190 for SS, and CP051261 for SI.

## Author Contributions

TS conceptualized the study. TL prepared the manuscript. TL, TS, and RM designed the experiment. TL, JK, and DA contributed to the laboratory work. RM, TS, DA, and GS reviewed and edited the manuscript. TS and RM supervised the study. GS contributed to the funding acquisition. All authors have read and agreed to the submitted version of the manuscript.

## Conflict of Interest

The authors declare that the research was conducted in the absence of any commercial or financial relationships that could be construed as a potential conflict of interest.

## Footnotes

1 https://github.com/rrwick/Porechop

2 https://github.com/rrwick/Filtlong

3 http://www.bx.psu.edu/~rsharris/lastz/

## Supplementary Material

FIGURE S1 Representative images of the *in vitro* bioprotection assay when challenging strain GW and BU with *Microdochium nivale*.

FIGURE S2 Representative images of the *in vitro* bioprotection assay when challenging strain SS and SI with *Microdochium nivale*.

TABLE S1 Pathogens used in the *in vitro* bioprotection assay.

TABLE S2 Programmed conditions of the glasshouse used in this study.

TABLE S3 *Xanthomonas* spp. genomes used in phylogeny and comparative genomics.

TABLE S4 Summary of reads available for genome assembly.

TABLE S5 The average nucleotide identity (ANI) between *Xanthomonas* spp. genomes used in comparative genomics.

TABLE S6 Pathogenicity-related gene clusters identified in *Xanthomonas* spp.
