# Supplementary material for: Novel Xanthomonas Species From the Perennial Ryegrass Seed Microbiome – Assessing the Bioprotection Activity of Non-pathogenic Relatives of Pathogens
Source: Front Microbiol. 2020 Aug 26;11:1991. doi: 10.3389/fmicb.2020.01991 (PMC7479056; doi:10.3389/fmicb.2020.01991)
Supplement: FIGURE S1 — Representative images of the in vitro bioprotection assay when challenging strain GW and BU with Microdochium nivale. [file Data_Sheet_1.docx]

**Novel *Xanthomonas* species from the perennial ryegrass seed microbiome - assessing the bioprotection activity of non-pathogenic relatives of pathogens**

**Supplementary Materials**

| Isolate | Rep 1 | Rep 2 | Rep 3 |
| --- | --- | --- | --- |
| Blank | 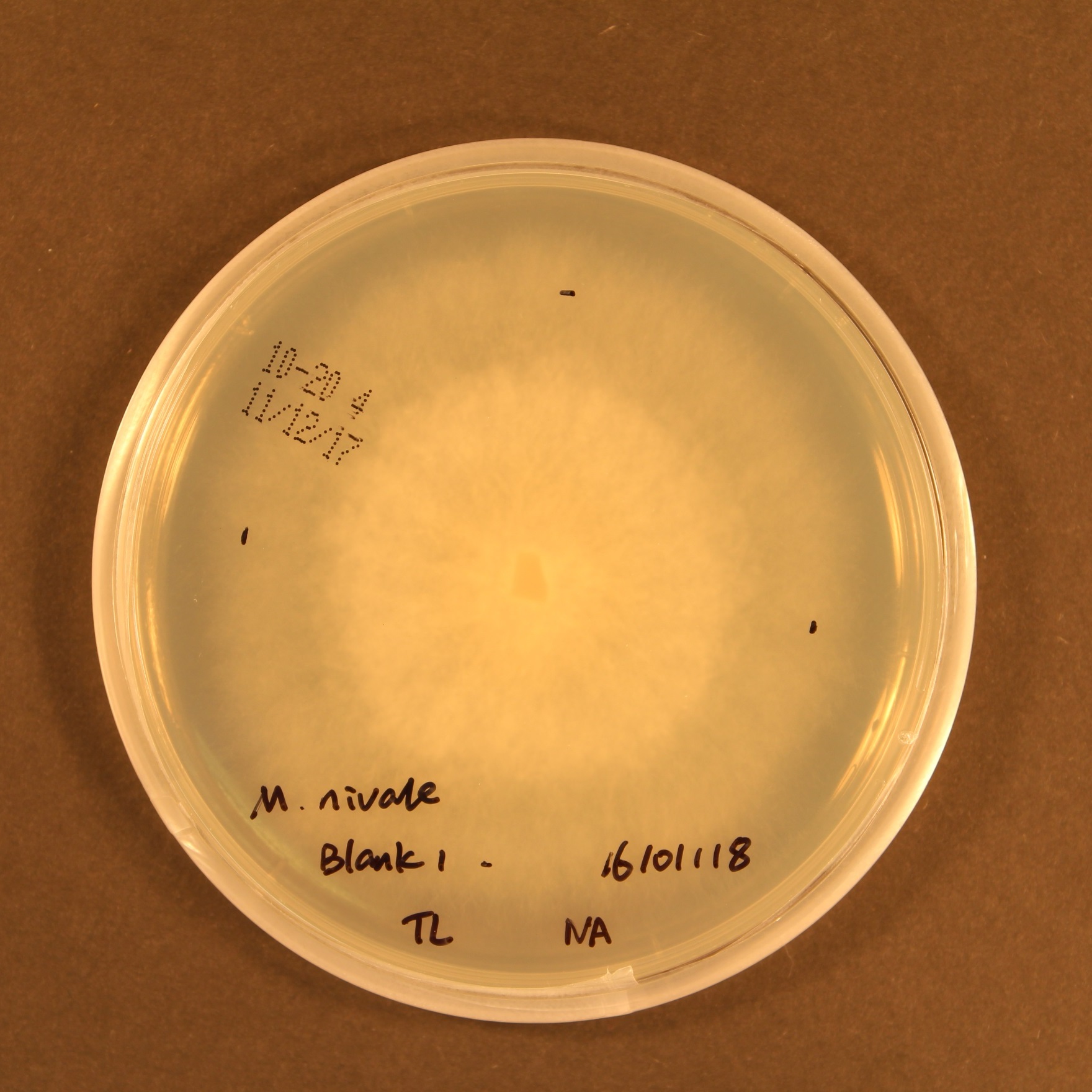 | 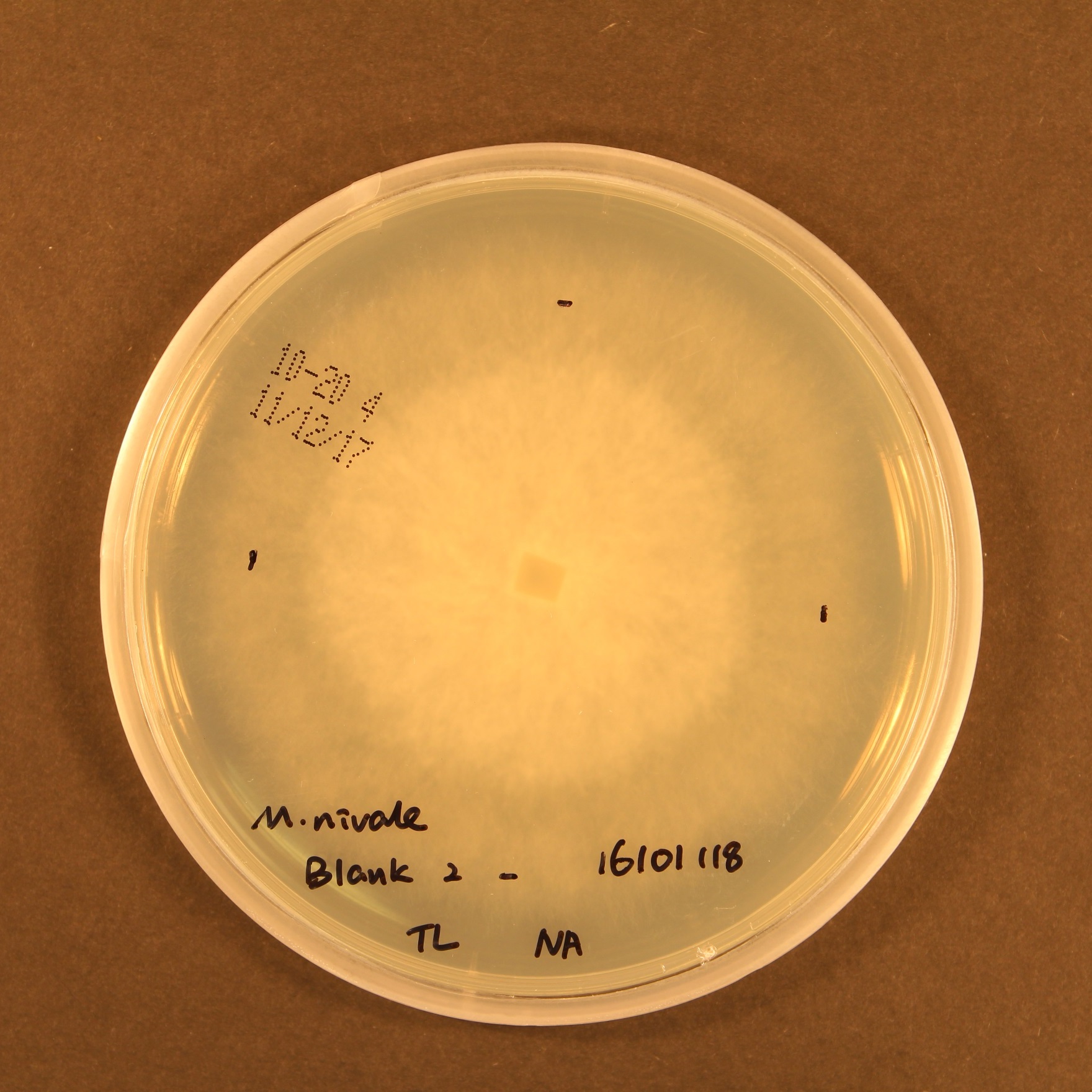 | 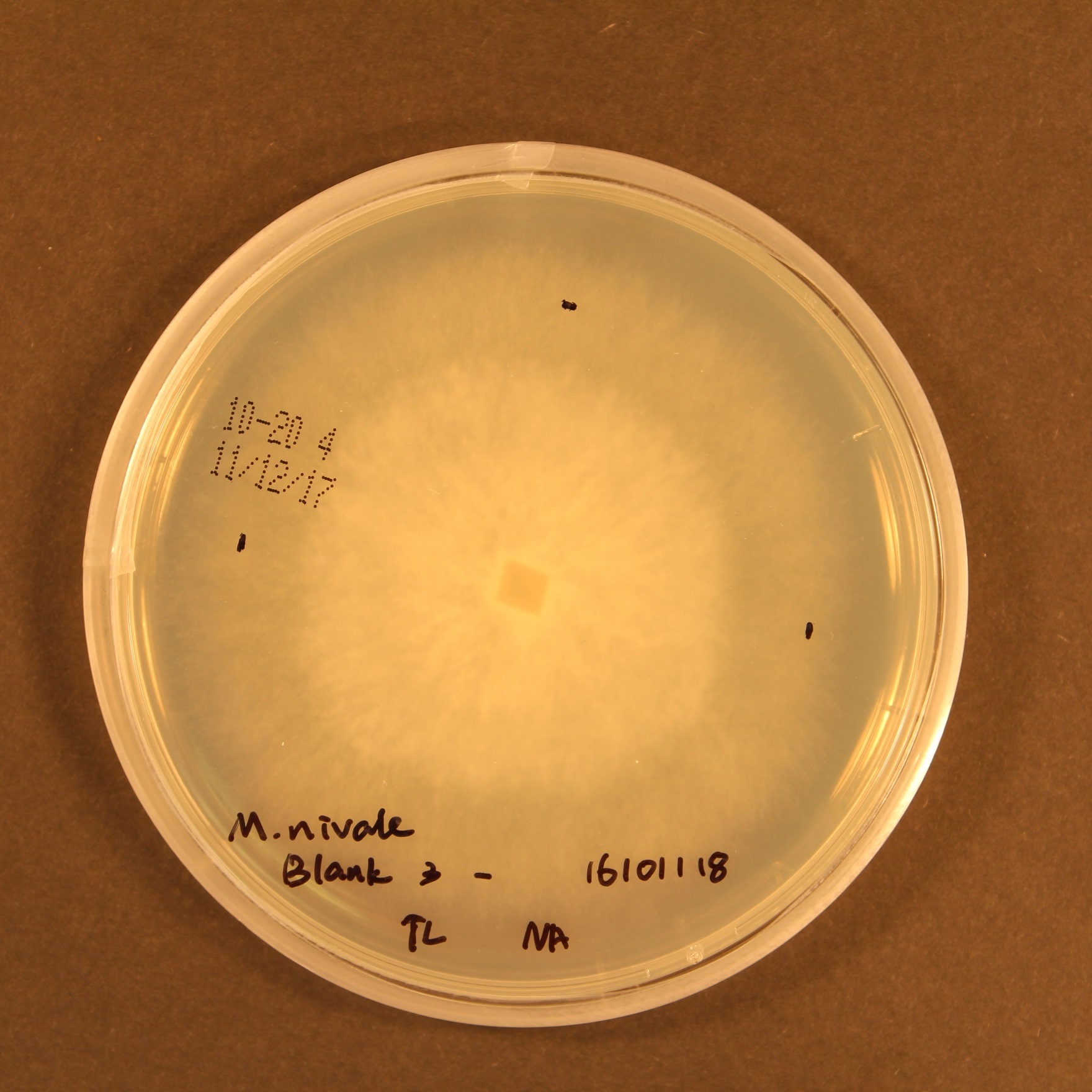 |
| GW | 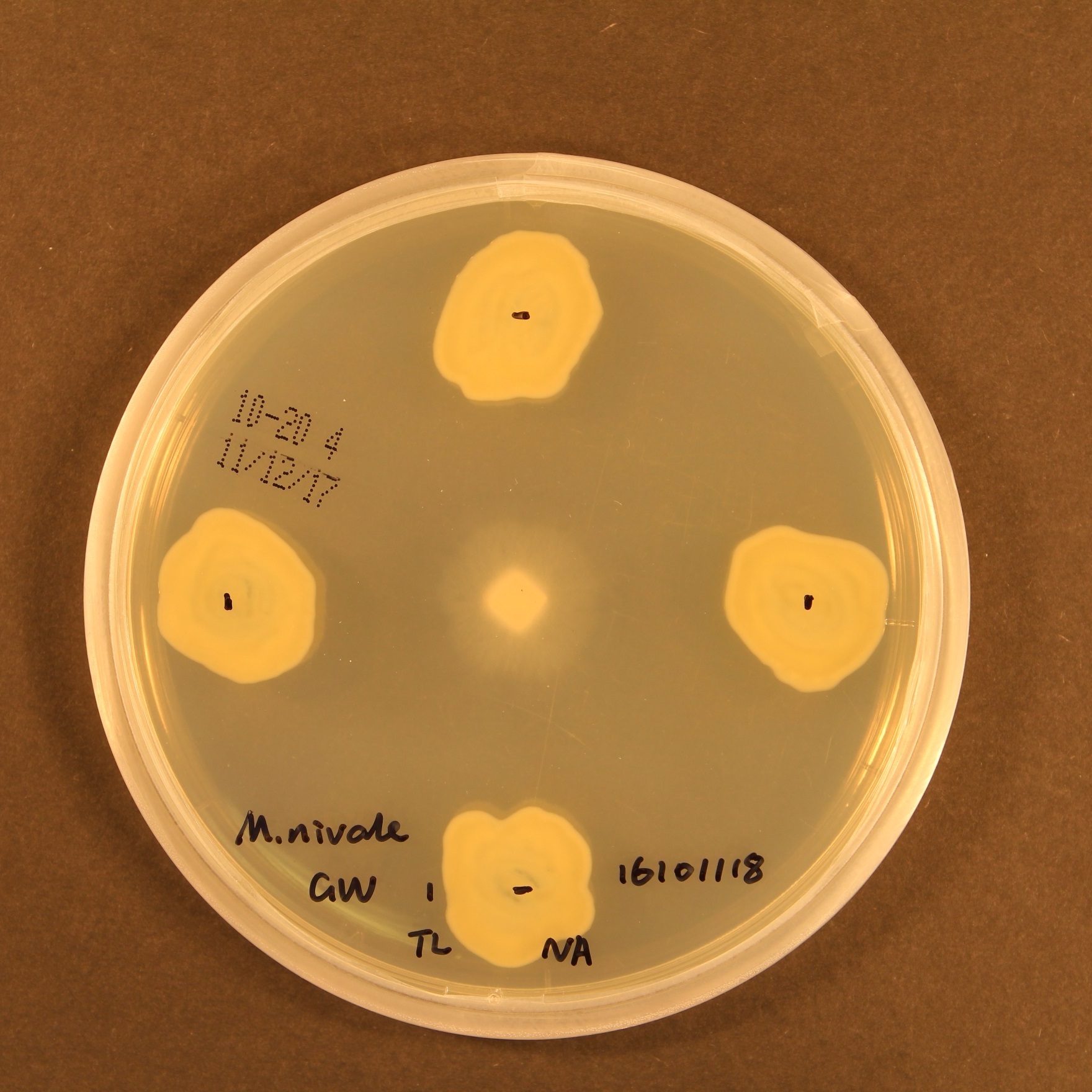 | 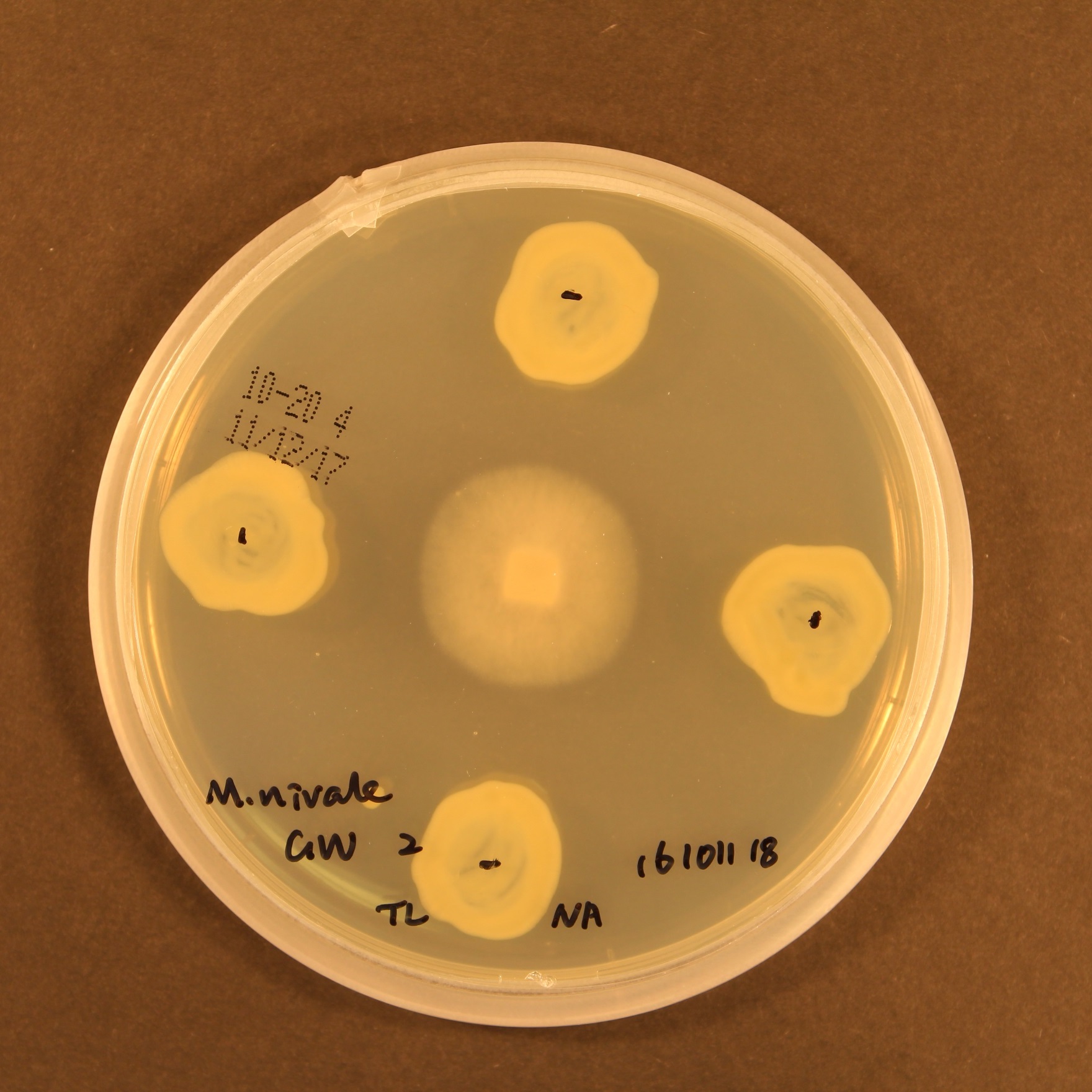 | 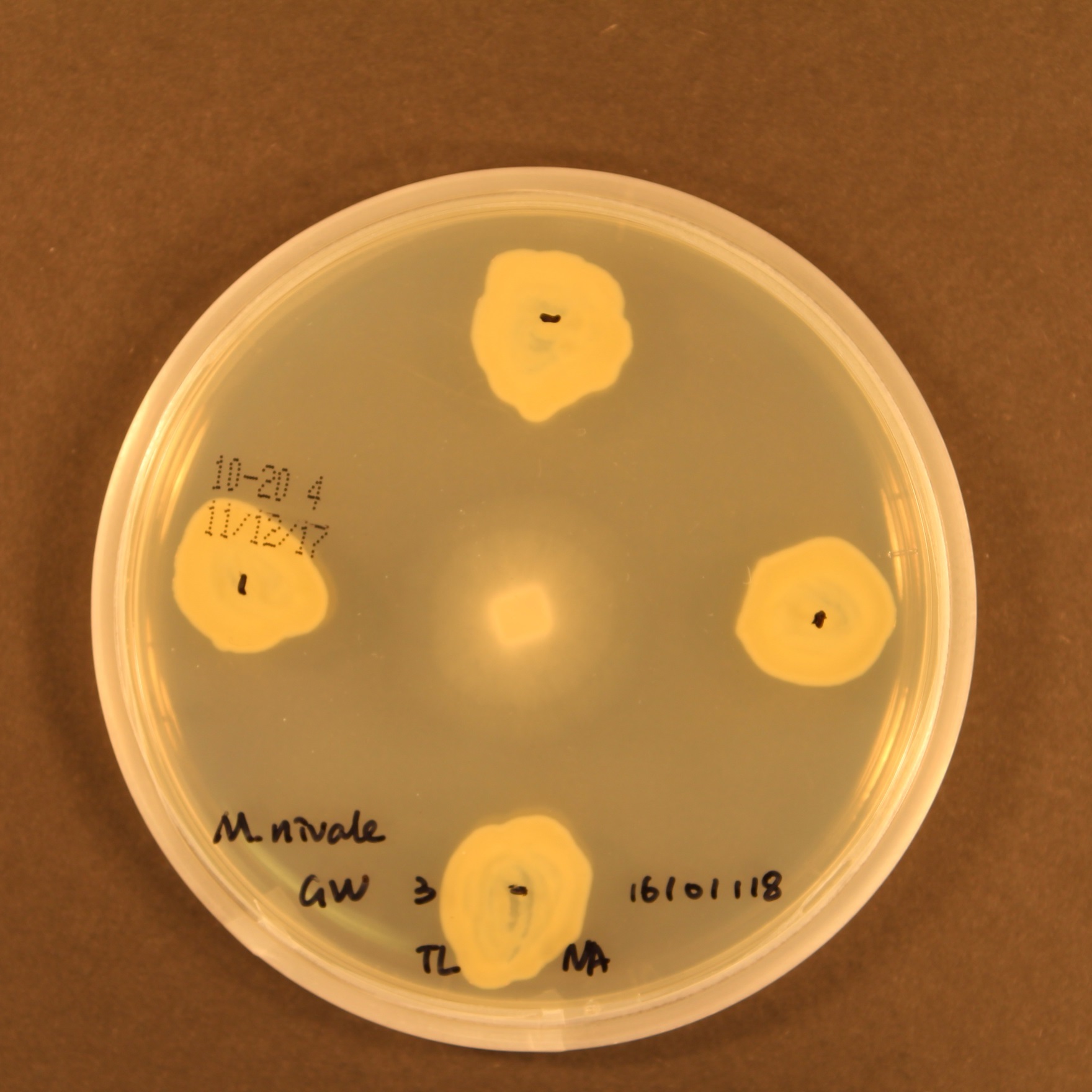 |
| BU | 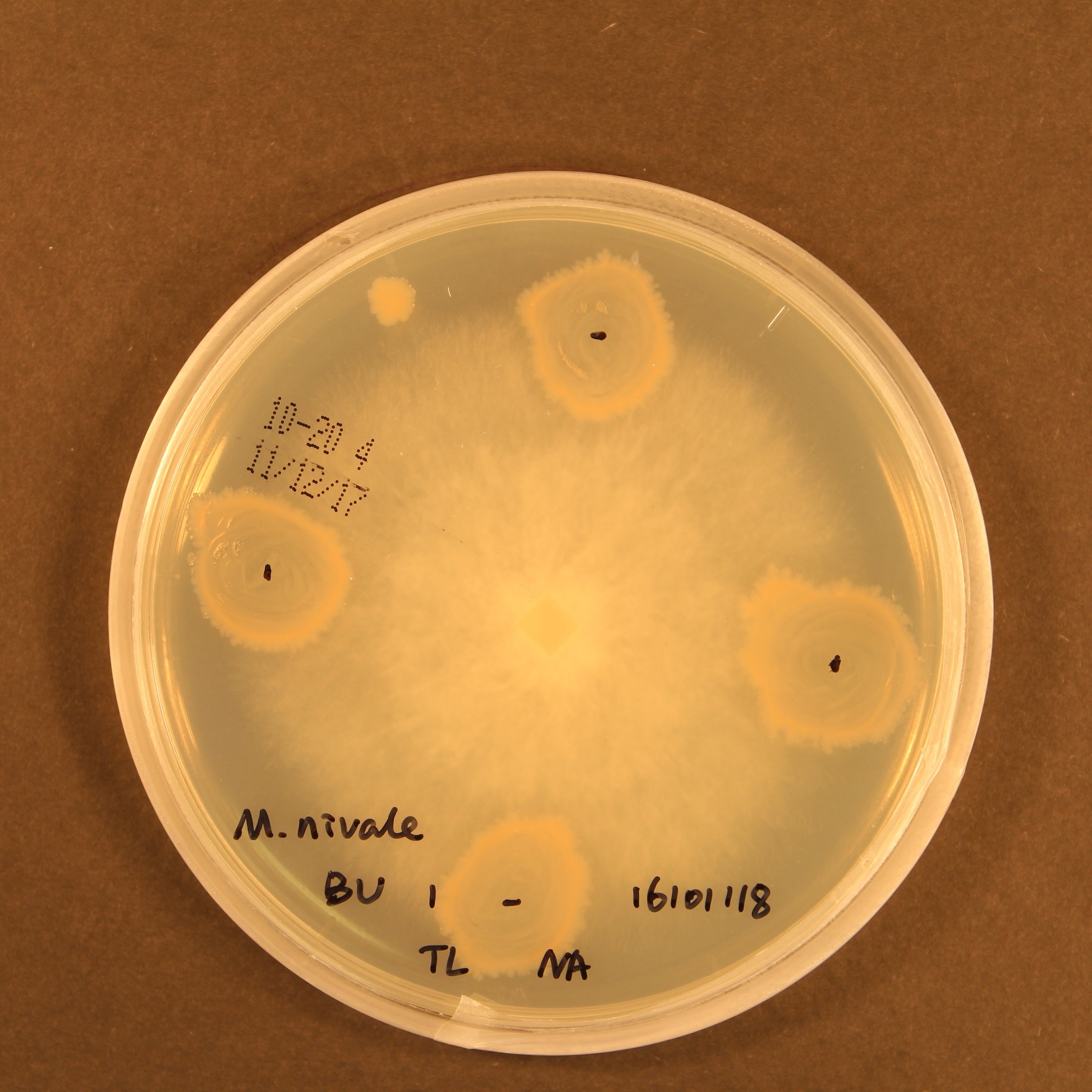 | 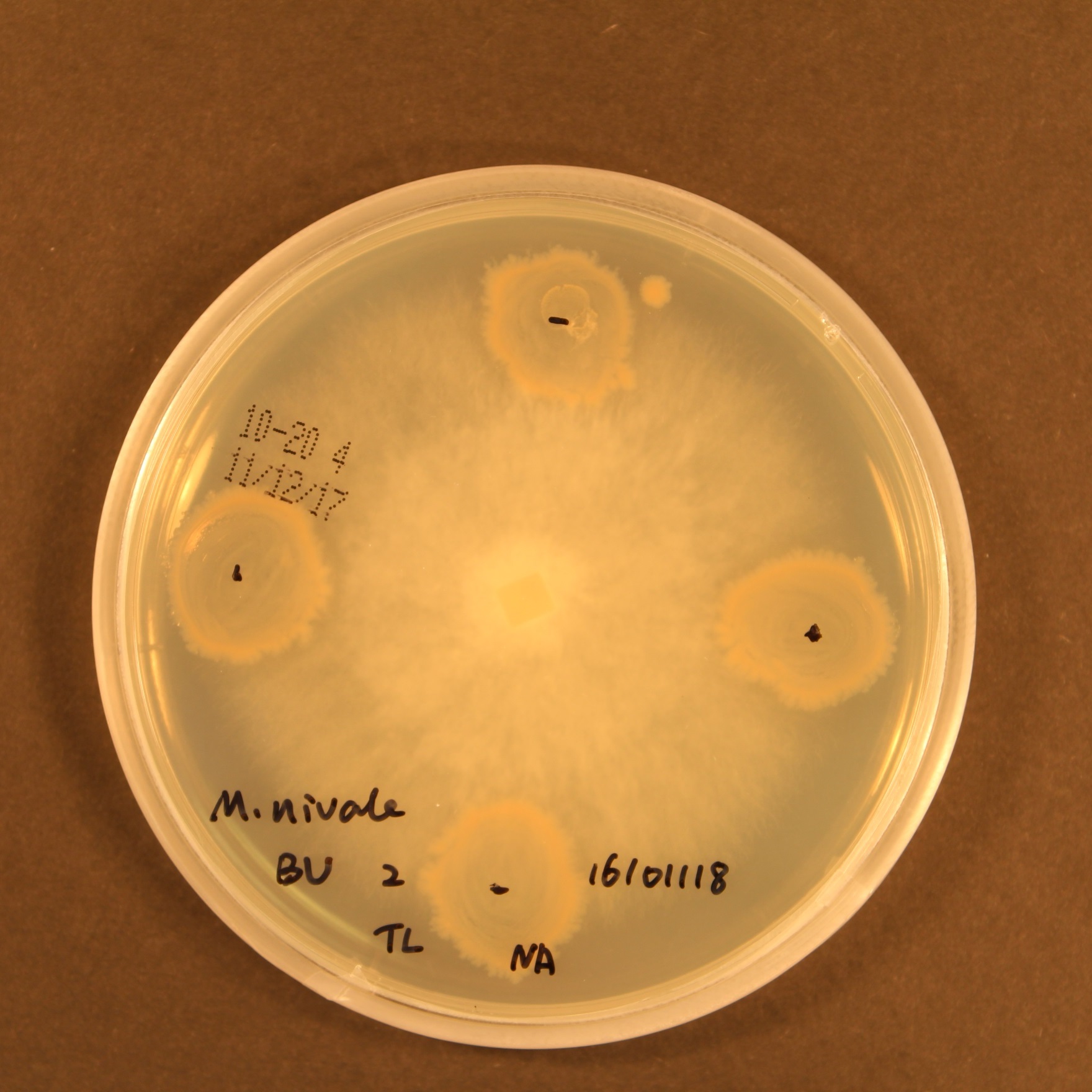 | 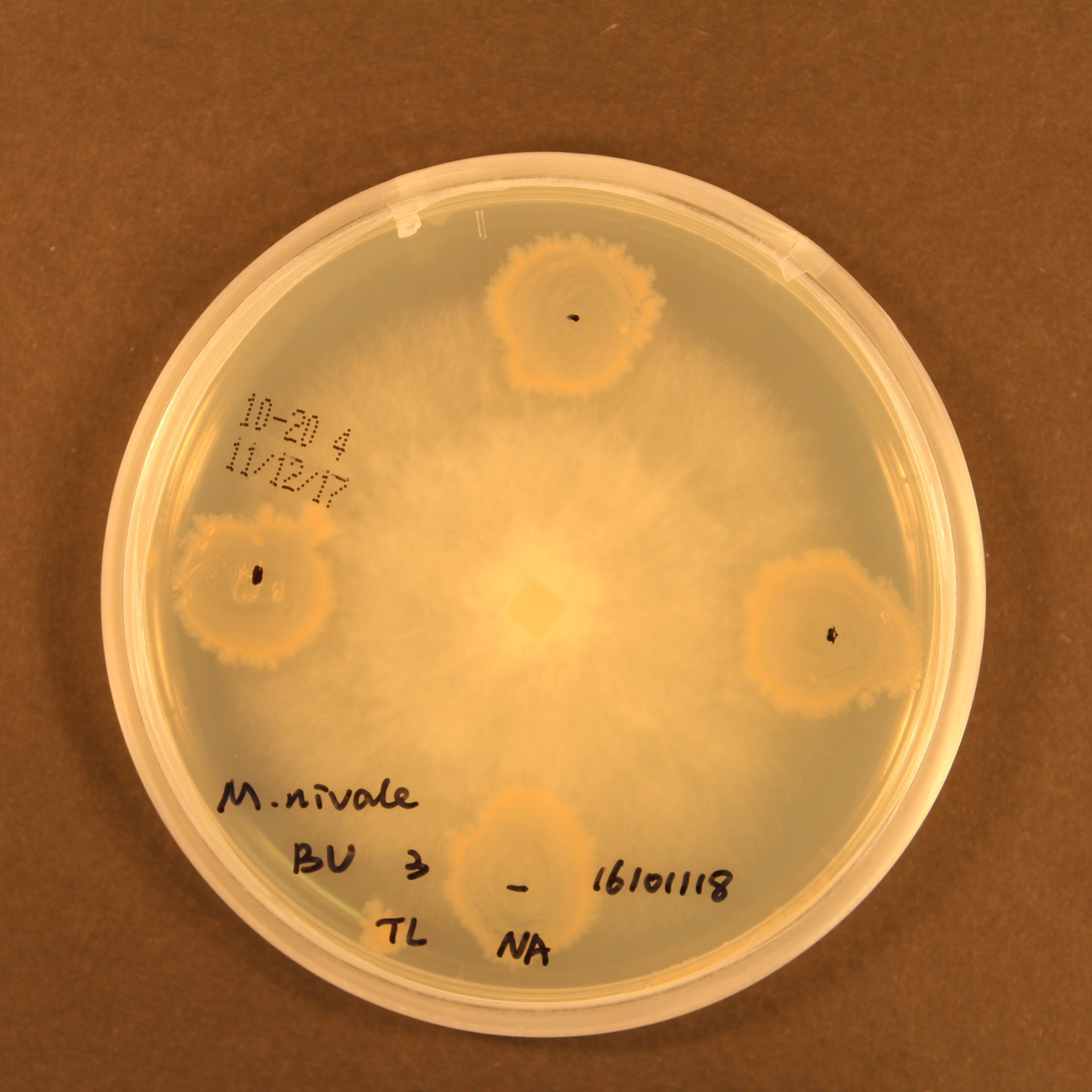 |

Figure S1. Representative images of the *in vitro* bioprotection assay when challenging strain GW and BU with *Microdochium nivale*.

| Isolate | Rep 1 | Rep 2 | Rep 3 |
| --- | --- | --- | --- |
| Blank | 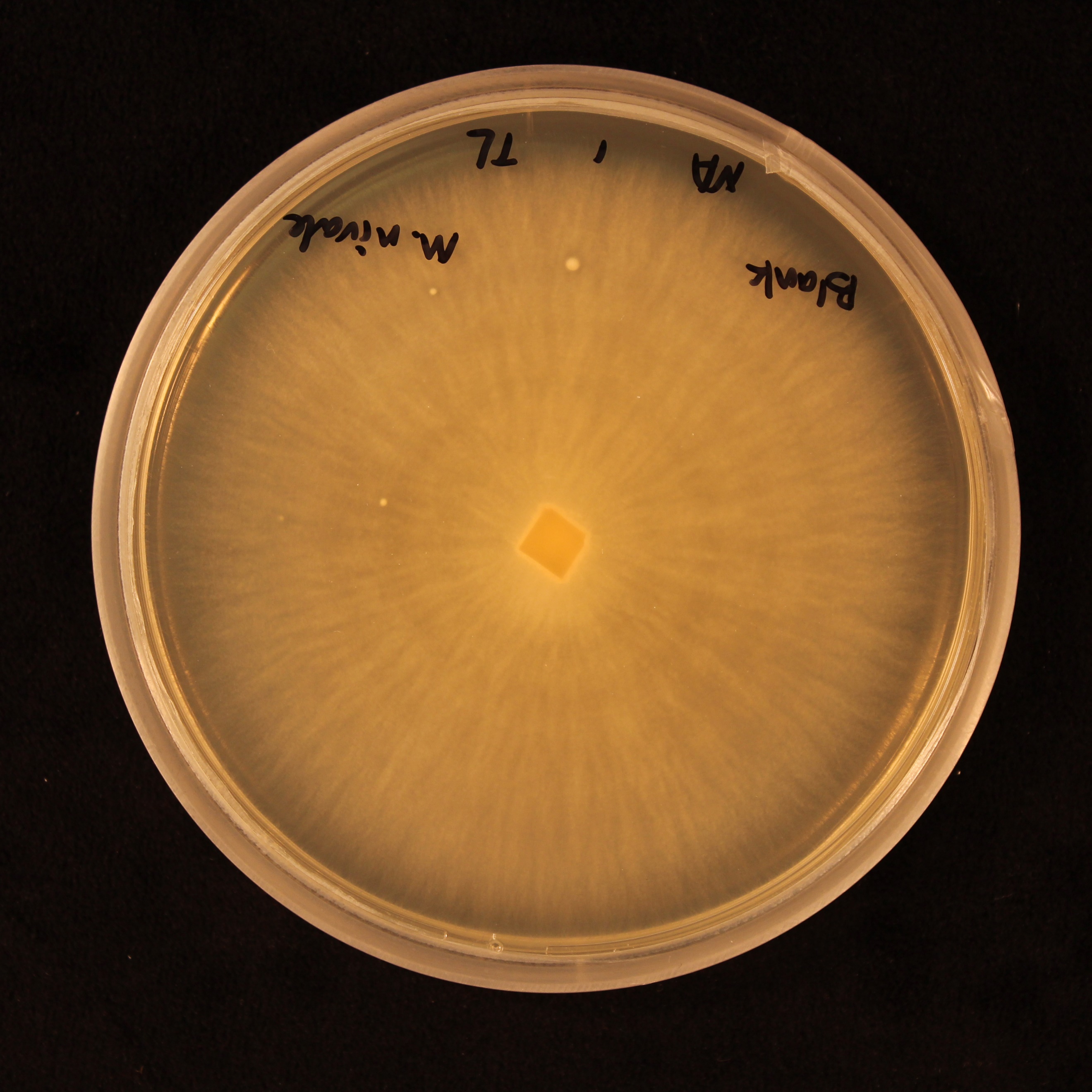 | 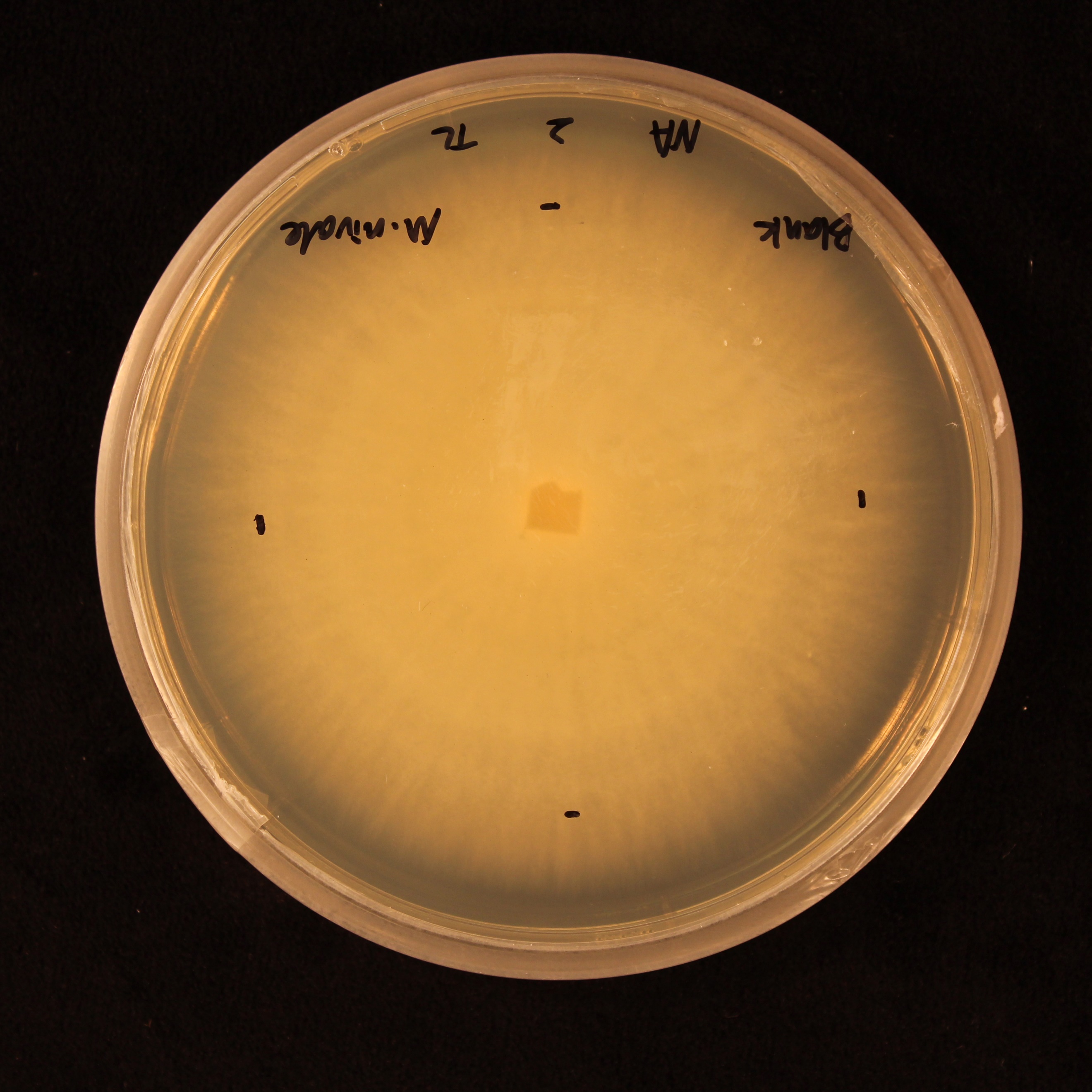 | 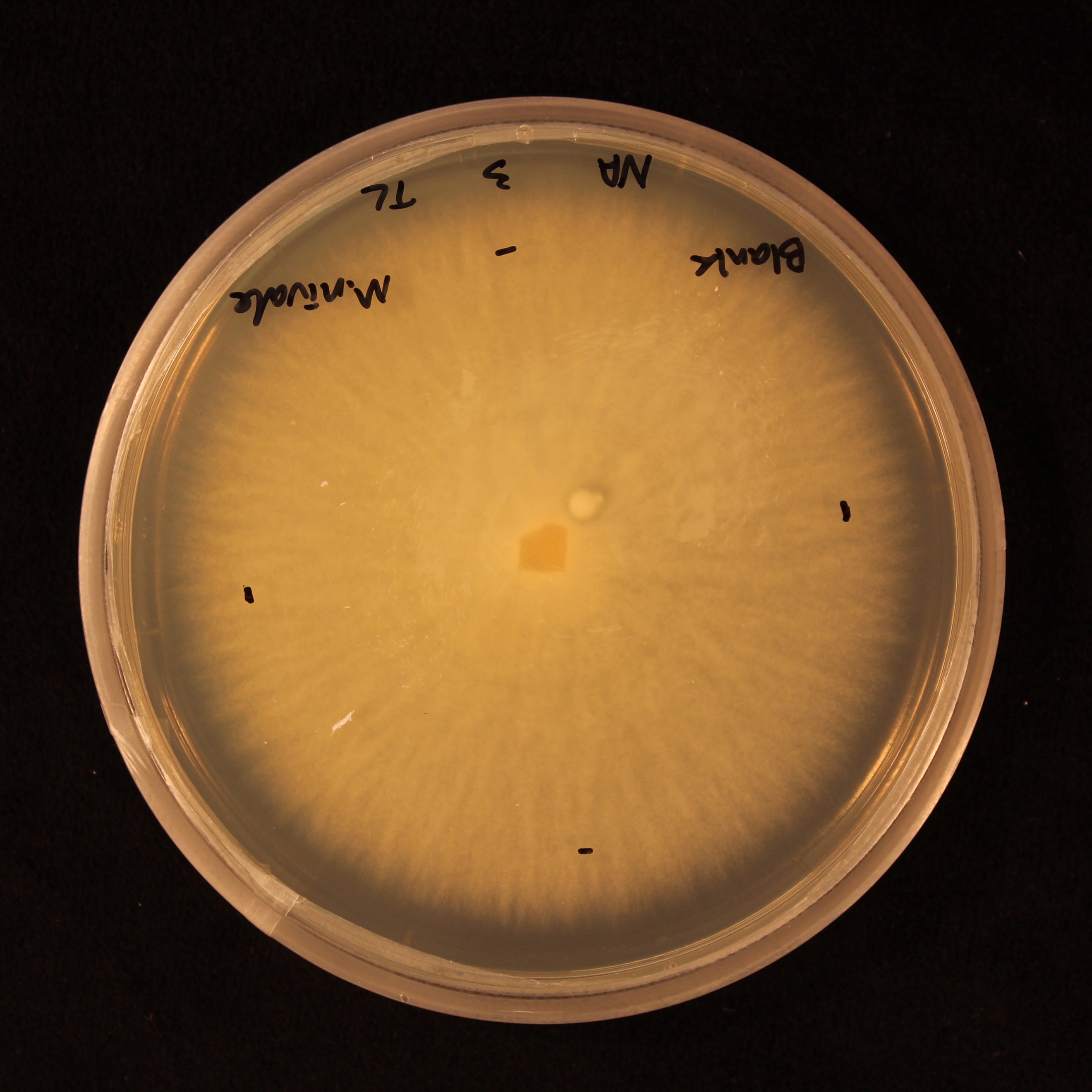 |
| SS | 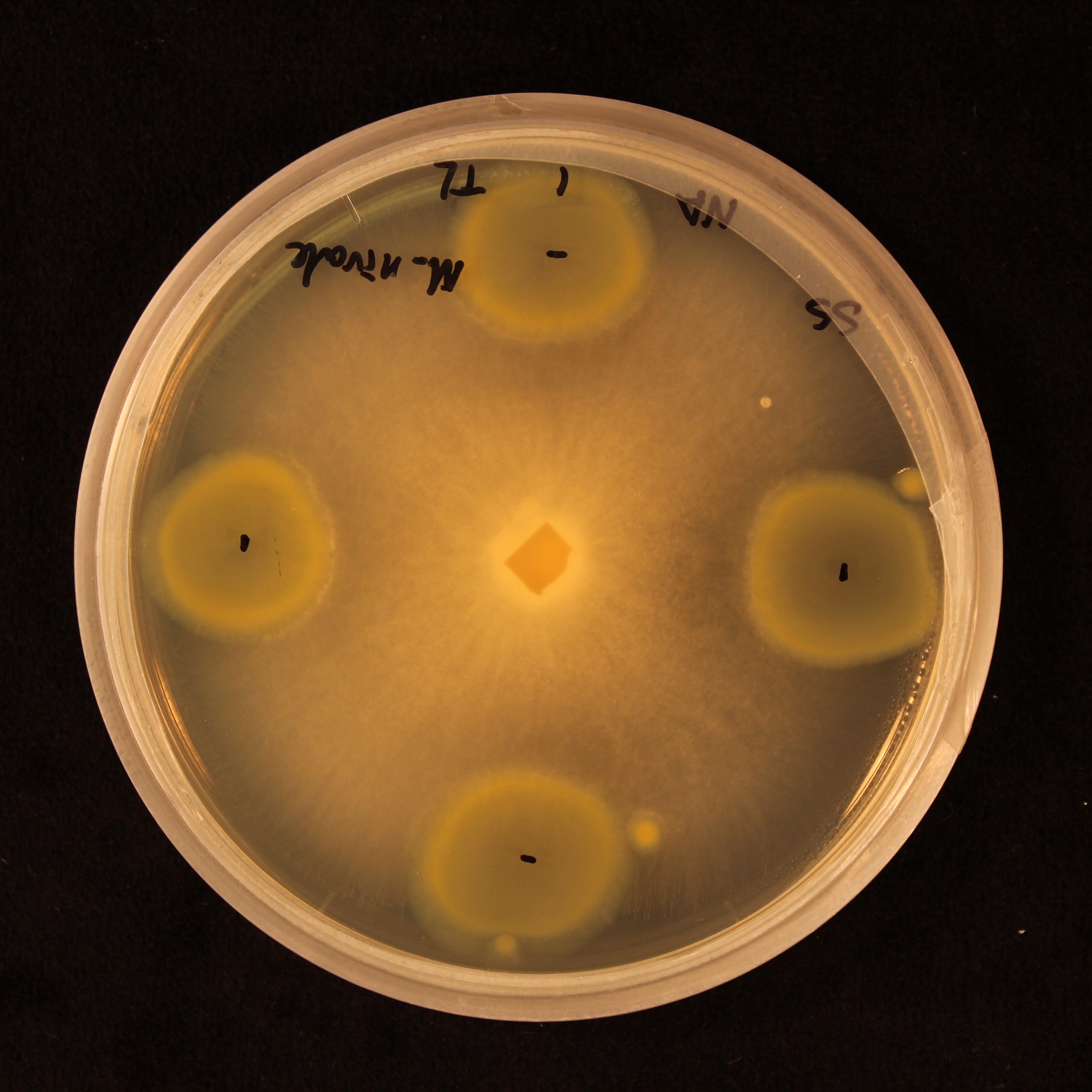 | 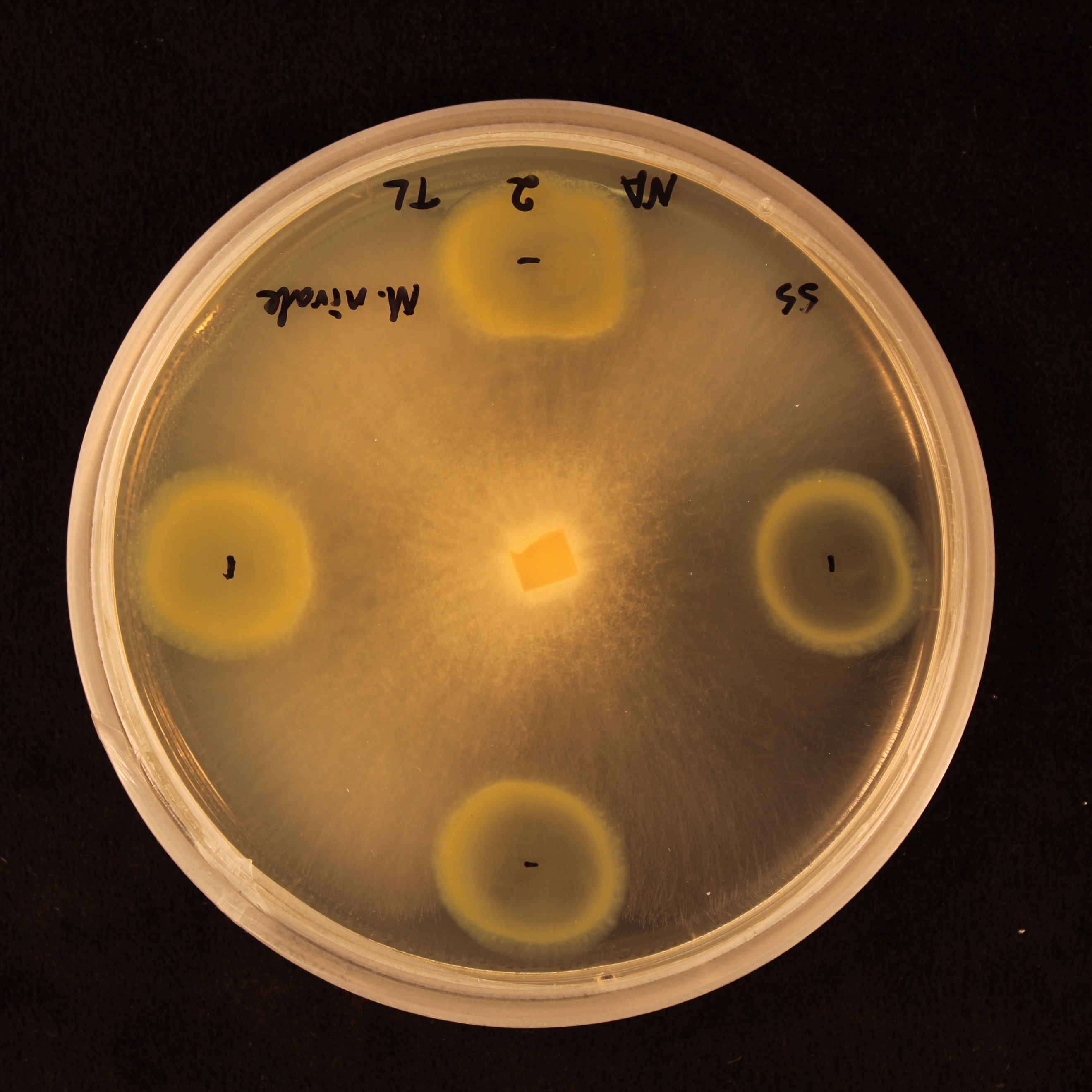 | 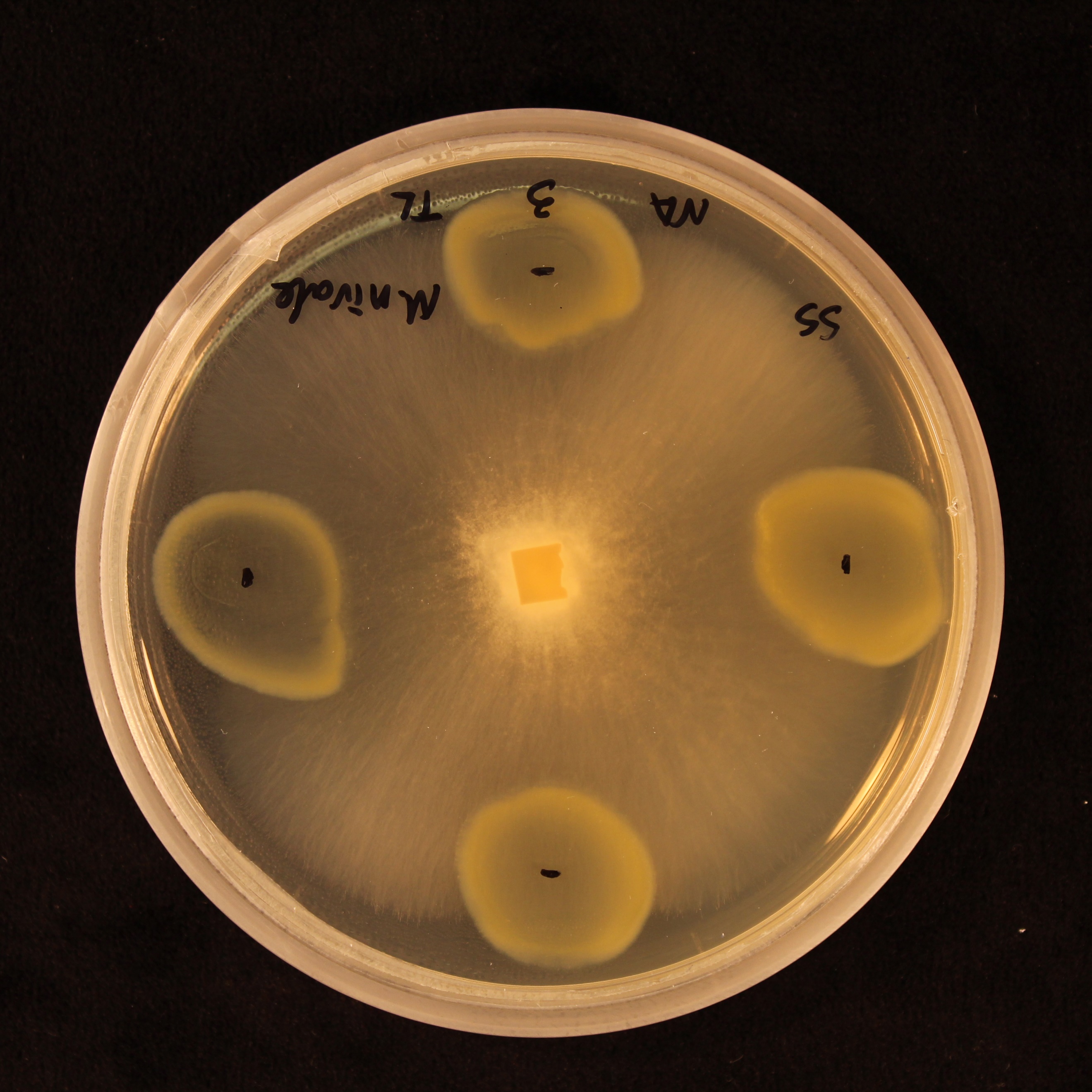 |
| SI | 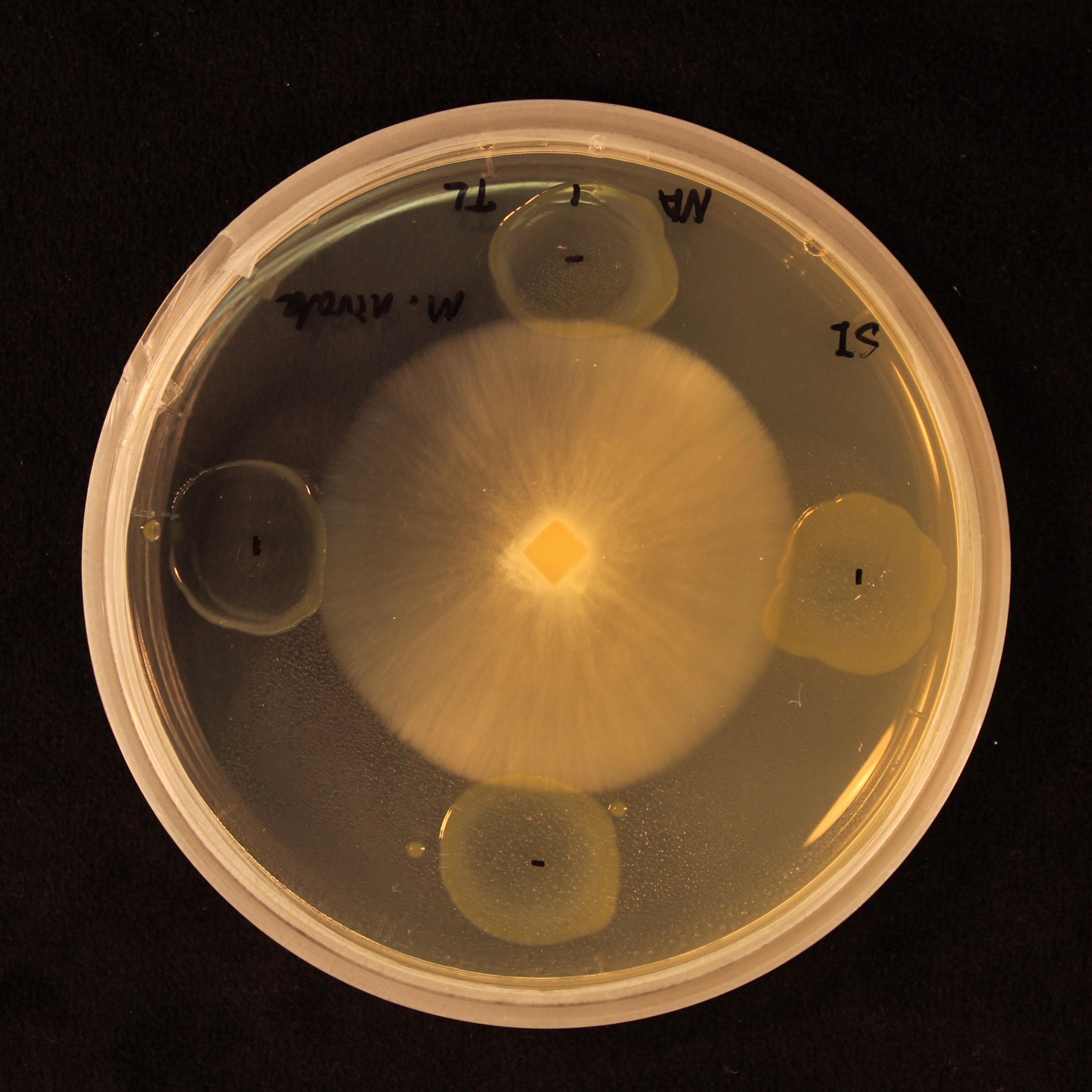 | 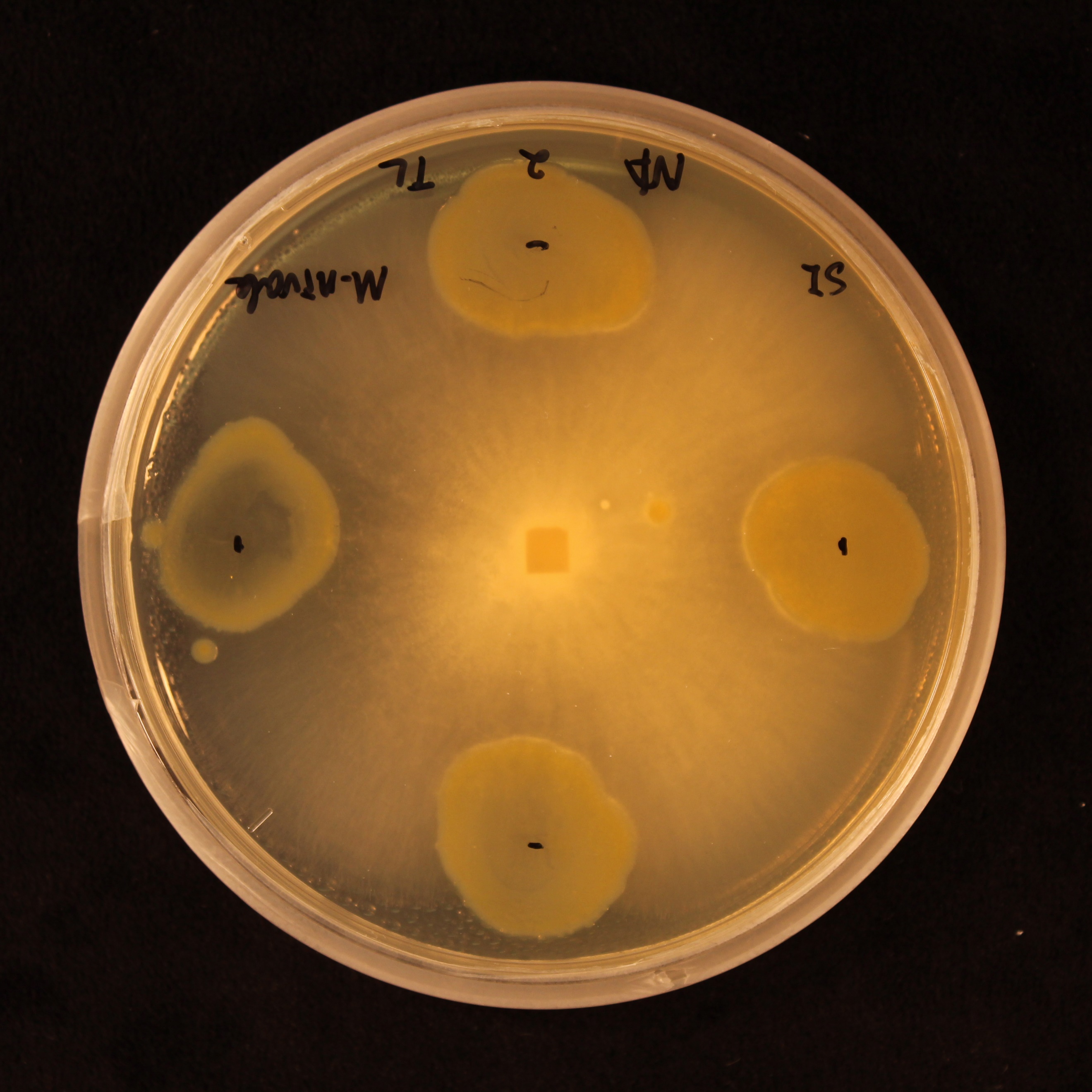 | 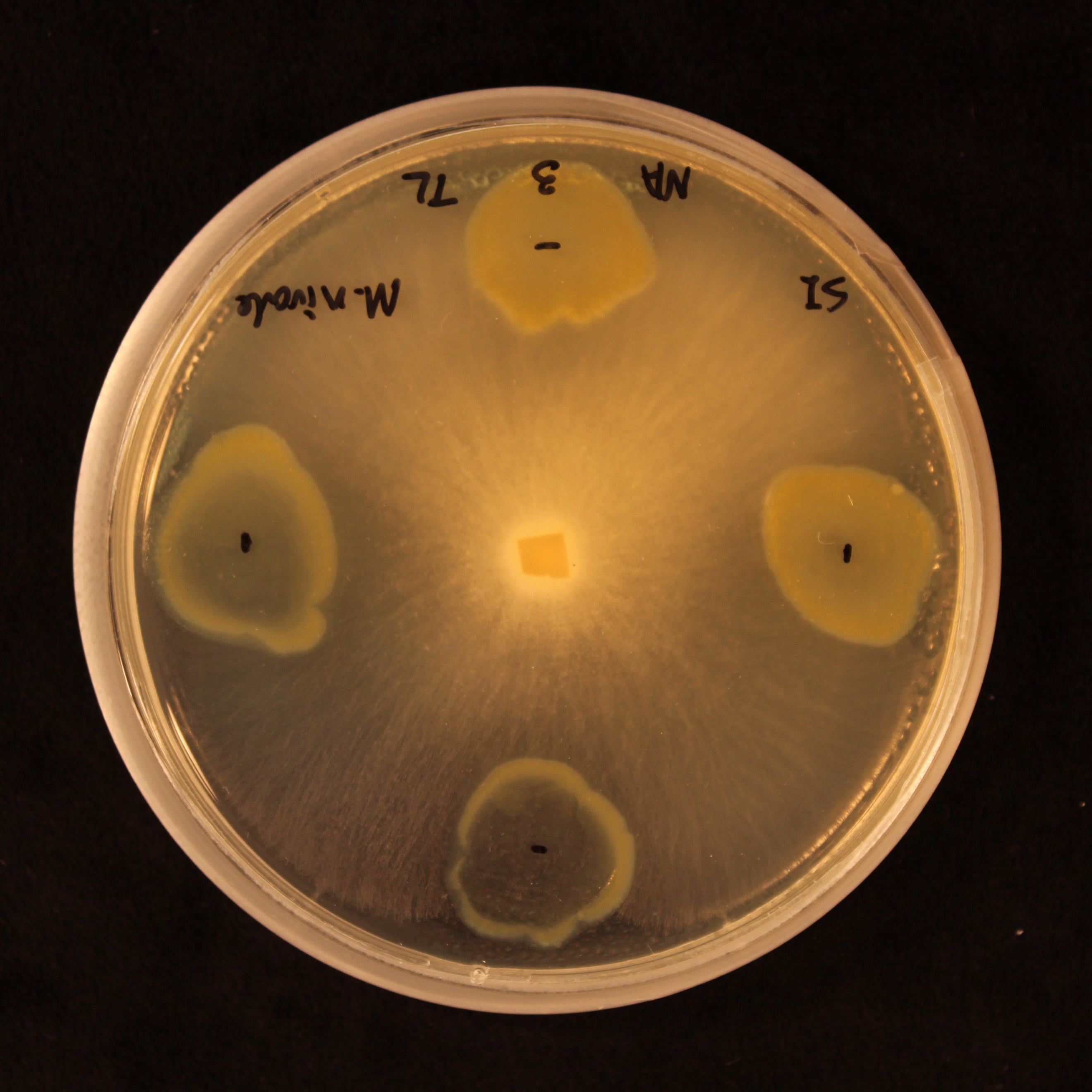 |

Figure S2. Representative images of the *in vitro* bioprotection assay when challenging strain SS and SI with *Microdochium nivale*.

Table S1. Pathogens used in the *in vitro* bioprotection assay.

| VPRI Acc. | Taxonomic Details | Host Taxonomic Details | State | Collection Date |
| --- | --- | --- | --- | --- |
| 12962 | *Drechslera brizae* (Y.Nisik.) Subram. & B.L.Jain | *Briza maxima* L. | Vic. | 24-Oct-85 |
| 32148 | *Sclerotium rolfsii* (Sacc.) | *Poa annua* L. | Vic. | 01-Jan-05 |
| 10694 | *Phoma sorghina* (Sacc.) Boerema, Dorenbosch, van Kesteren | *Cynodon dactylon* L*.* | Vic. | 19-Apr-79 |
| 42586a | *Fusarium verticillioides* (Sacc.) Nirenberg | *Zea mays* L. | Vic. | 27-Feb-15 |
| 42563 | *Bipolaris gossypina* | *Brachiaria* | Qld | N/A |
| 43403 | *Microdochium nivale* | *Lolium perenne* | Vic. | 11-Nov-17 |

Table S2. Programmed conditions of the glasshouse used in this study

| Day cycle | 6 am – 8 pm, 22 ℃  Growth lights will be turned on when outside light intensity drops below 170 W/m^2^. |
| --- | --- |
| Night cycle | 8 pm – 6 am, 14 ℃ |
| Relative humidity | around 50% to 60% |

Table S3. *Xanthomonas* spp. genomes used in phylogeny and comparative genomics (an excel file)

Table S4. Summary of reads available for genome assembly

| Strain ID | No. of bases (bp)  Illumina | No. of base (bp)  ONT | Mean length (bp)  ONT | Coverage  Illumina | Coverage  ONT |
| --- | --- | --- | --- | --- | --- |
| GW | 552,571,274 | 507,652,275 | 8,990 | 105.6× | 97× |
| SS | 6,055,588,549 | 123,011,703 | 9,299 | 1167.9× | 23.7× |
| SI | 3,066,769,952 | 130,414,053 | 12,411 | 584.5× | 24.9× |

Table S5. The average nucleotide identity (ANI) between *Xanthomonas* spp. genomes used in comparative genomics (an excel file)

Table S6. Pathogenicity-related gene clusters identified in *Xanthomonas* spp.

| Pathogenicity-related gene cluster | Reference | Presence in isolate GW |
| --- | --- | --- |
| T1SS | Lee et al. (2006) | Yes |
| T2SS | Lee et al. (2001), Filloux (2004) | Yes |
| T3SS | Wichmann et al. (2013) | No |
| T6SS | Boyer et al. (2009) | Yes |
| Type IV pilus | Dunger et al. (2016) | Yes |
| Flagellum | Darrasse et al. (2013) | Yes |
| Pathogenicity regulatory factors  (*rpf* gene cluster) | Tang et al. (1991) | Yes |
| Xanthan biosynthesis | Katzen et al. (1996), Vorhölter et al. (2008) | Yes |
| Lipopolysaccharide biosynthesis | Vorhölter et al. (2001) | Yes |

**REFERENCE**

Boyer, F., Fichant, G., Berthod, J., Vandenbrouck, Y., and Attree, I. (2009). Dissecting the bacterial type VI secretion system by a genome wide in silico analysis: what can be learned from available microbial genomic resources? *BMC Genomics* 10**,** 104.

Darrasse, A., Carrère, S., Barbe, V., Boureau, T., Arrieta-Ortiz, M.L., Bonneau, S., Briand, M., Brin, C., Cociancich, S., Durand, K., Fouteau, S., Gagnevin, L., Guérin, F., Guy, E., Indiana, A., Koebnik, R., Lauber, E., Munoz, A., Noël, L.D., Pieretti, I., Poussier, S., Pruvost, O., Robène-Soustrade, I., Rott, P., Royer, M., Serres-Giardi, L., Szurek, B., Van Sluys, M.-A., Verdier, V., Vernière, C., Arlat, M., Manceau, C., and Jacques, M.-a.J.B.G. (2013). Genome sequence of *Xanthomonas fuscans* subsp. *fuscansstrain* 4834-R reveals that flagellar motility is not a general feature of xanthomonads *BMC Genomics* 14**,** 761.

Dunger, G., Llontop, E., Guzzo, C.R., and Farah, C.S. (2016). The *Xanthomonas* type IV pilus. *Curr Opin Microbiol* 30**,** 88-97.

Filloux, A. (2004). The underlying mechanisms of type II protein secretion. *Biochimica et Biophysica Acta (BBA)-Molecular Cell Research* 1694**,** 163-179.

Katzen, F., Becker, A., Zorreguieta, A., Pühler, A., and Ielpi, L.J.J.O.B. (1996). Promoter analysis of the *Xanthomonas campestris* pv. *campestris* gum operon directing biosynthesis of the xanthan polysaccharide *J Bacteriol* 178**,** 4313-4318.

Lee, H.M., Tyan, S.W., Leu, W.M., Chen, L.Y., Chen, D.C., and Hu, N.T. (2001). Involvement of the XpsN protein in formation of the XpsL-xpsM complex in *Xanthomonas campestris* pv. *campestris* type II secretion apparatus. *J Bacteriol* 183**,** 528-535.

Lee, S.-W., Han, S.-W., Bartley, L.E., and Ronald, P.C. (2006). Unique characteristics of *Xanthomonas oryzae* pv. *oryzae* AvrXa21 and implications for plant innate immunity. *Proc Natl Acad Sci* 103**,** 18395-18400.

Tang, J.-L., Liu, Y.-N., Barber, C.E., Dow, J.M., Wootton, J.C., Daniels, M.J.J.M., and Mgg, G.G. (1991). Genetic and molecular analysis of a cluster of rpf genes involved in positive regulation of synthesis of extracellular enzymes and polysaccharide in *Xanthomonas campestris* pathovar *campestris. Molecular and General Genetics MGG* 226**,** 409-417.

Vorhölter, F.-J., Niehaus, K., and Pühler, A. (2001). Lipopolysaccharide biosynthesis in *Xanthomonas campestris* pv. *campestris* : a cluster of 15 genes is involved in the biosynthesis of the LPS O-antigen and the LPS core. *Molecular Genetics and Genomics* 266**,** 79-95.

Vorhölter, F.-J., Schneiker, S., Goesmann, A., Krause, L., Bekel, T., Kaiser, O., Linke, B., Patschkowski, T., Rückert, C., and Schmid, J. (2008). The genome of *Xanthomonas campestris* pv. *campestris* B100 and its use for the reconstruction of metabolic pathways involved in xanthan biosynthesis. *Journal of biotechnology* 134**,** 33-45.

Wichmann, F., Vorhӧlter, F.J., Hersemann, L., Widmer, F., Blom, J., Niehaus, K., Reinhard, S., Conradin, C., and Kӧlliker, R. (2013). The noncanonical type III secretion system of *Xanthomonas translucens* pv. *graminis* is essential for forage grass infection. *Mol Plant Pathol* 14**,** 576-588.
